# Differential Assembly of Catalytic Interactions within the Conserved Active Sites of Two Ribozymes

**DOI:** 10.1371/journal.pone.0160457

**Published:** 2016-08-08

**Authors:** Sabine N. S. van Schie, Raghuvir N. Sengupta, Daniel Herschlag

**Affiliations:** 1 Department of Biochemistry, Stanford University, Stanford, California, 94305, United States of America; 2 Leiden Institute of Chemistry, Leiden University, Leiden, 2333 CC, the Netherlands; 3 Departments of Chemical Engineering and Chemistry, Stanford University, Stanford, California, 94305, United States of America; 4 Stanford ChEM-H (Chemistry, Engineering, and Medicine for Human Health), Stanford University, Stanford, California, 94305, United States of America; Oak Ridge National Laboratory, UNITED STATES

## Abstract

Molecular recognition is central to biology and a critical aspect of RNA function. Yet structured RNAs typically lack the preorganization needed for strong binding and precise positioning. A striking example is the group I ribozyme from *Tetrahymena*, which binds its guanosine substrate (G) orders of magnitude slower than diffusion. Binding of G is also thermodynamically coupled to binding of the oligonucleotide substrate (S) and further work has shown that the transition from E•G to E•S•G accompanies a conformational change that allows G to make the active site interactions required for catalysis. The group I ribozyme from *Azoarcus* has a similarly slow association rate but lacks the coupled binding observed for the *Tetrahymena* ribozyme. Here we test, using G analogs and metal ion rescue experiments, whether this absence of coupling arises from a higher degree of preorganization within the *Azoarcus* active site. Our results suggest that the *Azoarcus* ribozyme forms cognate catalytic metal ion interactions with G in the E•G complex, interactions that are absent in the *Tetrahymena* E•G complex. Thus, RNAs that share highly similar active site architectures and catalyze the same reactions can differ in the assembly of transition state interactions. More generally, an ability to readily access distinct local conformational states may have facilitated the evolutionary exploration needed to attain RNA machines that carry out complex, multi-step processes.

## Introduction

Molecular recognition is essential for RNA function, allowing structured RNAs to bind and sometimes transform a diverse array of ligands, including nucleic acids, proteins, and small molecules [1–4] with high specificity. Nevertheless, RNA functional sites are often limited in preorganization, perhaps a reflection of RNA’s tendency to form alternative interactions [5, 6], and often require conformational rearrangements to accommodate bound ligands (e.g. [1, 7–11]). A rich landscape of accessible RNA conformational states may have been harnessed by Nature through natural selection to coordinate complex, multistep processes such as protein synthesis and splicing and to regulate gene expression (e.g. [12–16]).

The *Tetrahymena* group I intron provides a classic example of limited preorganization within an RNA functional site. This ribozyme (E) catalyzes cleavage of an oligonucleotide substrate (S) by an exogenous guanosine cofactor (G) [17]. G binding to the *Tetrahymena* ribozyme is extraordinarily slow, (~10^5^ M^-1^ min^-1^ [8]), orders of magnitude below the diffusion limit, indicating that the G binding site exists predominantly in one or more states that do not allow G binding [8, 18, 19], and additional experiments [8] provided evidence for a transient opening, with a rate constant of ~10^2^–10^3^ s^-1^, to a state that is competent to bind G.

**Fig 1:**
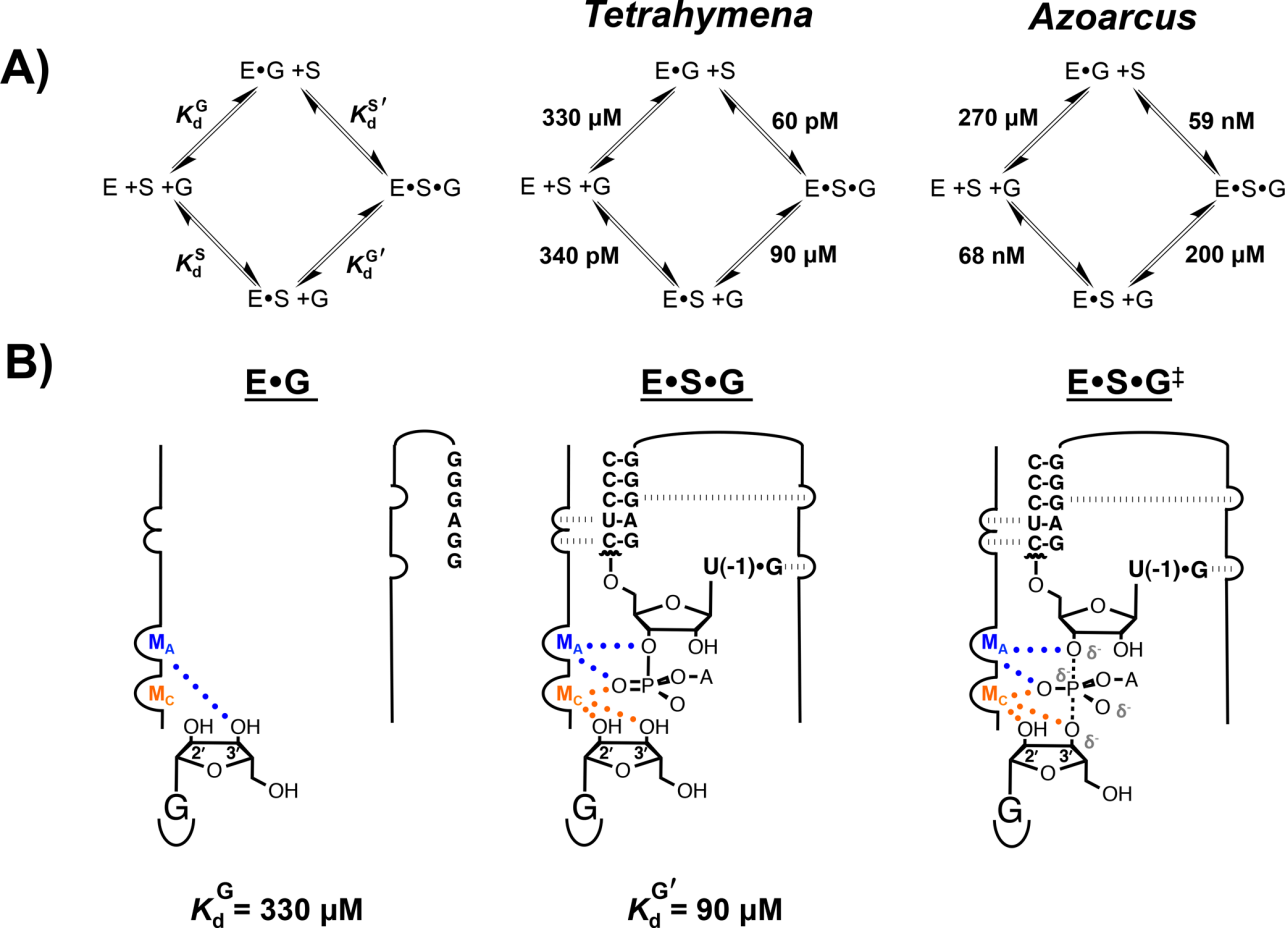
The group I ribozyme reaction. (A) General framework for the group I ribozyme reaction (left) presenting equilibrium dissociation constants of the G and S substrates to the *Tetrahymena* (middle, [20, 22]) and *Azoarcus* (right, [23]) ribozymes. (B) Model for the assembly of catalytic metal ion interactions with M_A_ (blue) and M_C_ (orange) in the *Tetrahymena* group I ribozyme active site [19, 21, 24]. Closed dots and hatched lines represent metal ion interaction and hydrogen bonds, respectively. Partial negative charges are represented by ‘δ^-^’. Prior data suggest that a third metal ion, M_B_, contacts the G 3′-oxygen atom instead of M_C_ in the transition state [25] but this interaction is likely an artifact from functional experiments, as described in the Discussion and elsewhere [19]. For simplicity, we show M_C_ contacting the G 3′-oxygen atom in the transition state.

Once G is bound, a second rearrangement occurs to assemble the active site metal ion interactions required for catalysis [19]. Initial evidence for this conformational change came from the observation that binding of G and S to the ribozyme is cooperative, with the affinity of G approximately 4-fold stronger to E•S (KdG' = 90 μM) than to E ($K_{\text{d}}^{\text{G}}$ = 330 μμM) (Fig 1A, [20]). More recently, this coupling was shown to accompany an active site rearrangement that enables the nucleophilic G 3′-OH group and the adjacent 2′-OH to contact an active site metal ion, termed M_C_, within E•S•G (Fig 1B, [19, 21]).

The interactions between M_C_ and the 2′- and 3′-OH groups of G in the E•S•G complex are made subsequent to this rearrangement and are likely retained in the chemical step (Fig 1B, [19]). In contrast, in the absence of bound S, G assumes a functionally inactive “off-pathway” configuration, with its 3′-OH group contacting a different metal ion, termed M_A_, and the adjacent 2′-OH making no interaction (Fig 1B). Structural analysis and molecular modeling suggest that binding of S promotes a structural rearrangement that repositions the two active site metal ions (M_A_ and M_C_) and alters the conformation of the G ribose ring [19].

A recent kinetic and thermodynamic reaction framework of the *Azoarcus* group I ribozyme provides an opportunity to compare and contrast functional site preorganization between the *Tetrahymena* and *Azoarcus* ribozymes [23]. Like the *Tetrahymena* ribozyme, binding of G to the *Azoarcus* ribozyme is slow, ~10^4^−10^5^ M^-1^ min^-1^, suggesting that the G binding site of *Azoarcus* also favors one or more alternative states that are unable to bind G. However, in contrast to *Tetrahymena*, the affinity of G to E and E•S for the *Azoarcus* ribozyme are similar ($K_{\text{d}}^{\text{G}}$ = 270 and KdG' = 200 μM, Fig 1A), suggesting most simply that interactions made with G are the same with and without bound S. Alternatively, the absence of coupling between G and S for the *Azoarcus* ribozyme may be an energetic coincidence.

Here we distinguish between these models by probing active site interactions with G in the *Azoarcus* E•G and E•S•G complexes. Our results are consistent with the model in which catalytic metal ion interactions between M_C_ and the G 2′- and 3′-OH groups are made in the *Azoarcus* E•G complex and remain unchanged in the subsequent E•S•G complex. These results suggest differences in active site organization between the *Azoarcus* and *Tetrahymena* ribozymes, despite sharing highly similar active site architectures.

## Results

To probe active site interactions with G in the E•G and E•S•G complexes, we utilized metal ion rescue experiments, which rely on the preferred coordination of soft metal ions to sulfur and nitrogen compared to oxygen (e.g. [17, 25–28]). Prior rescue experiments with the *Tetrahymena* ribozyme provided evidence for metal ion interactions in the transition state, with two active site metal ions, M_A_ and M_C_, contacting the G 2′- and 3′-oxygen atoms, the U(-1) 3′-oxygen atom on S and the reactive phosphoryl group (Fig 1B, [25, 29]). In addition, recent rescue experiments indicate that the G 2′- and 3′-OH groups, which likely contact M_C_ in the transition state, make these interactions in E•S•G, but make non-catalytic interactions in E•G, as described above and depicted in Fig 1B [19].

The crystallographic data for the *Azoarcus* ribozyme show two active site metal ions analogous to M_A_ and M_C_, with M_C_ within coordination distance to the G 2′- and 3′-OH groups when S is present in the active site [30]. These apparent contacts are equivalent to interactions deduced from metal ion rescue experiments with the *Tetrahymena* ribozyme in the E•S•G complex (Fig 1B, [19]). X-ray structures of the *Azoarcus* E•G complex are not available but the reaction framework shown in Fig 1A indicates that G has the same binding affinity in the presence and absence of S. This observation, most simply, suggests that the catalytic metal ion interactions between M_C_ and the G -OH groups in the E•S•G complex are also made in the E•G complex. Alternatively, G may assume a different configuration within the *Azoarcus* E•G complex, like the *Tetrahymena* ribozyme (Fig 1B, [19]), with the affinity of G to E and E•S being coincidentally the same.

To distinguish these models, we replaced the G 2′- and 3′-OH groups with an amino group (-NH_2_) and measured binding of 2′- and 3′-aminoguanosine to the *Azoarcus* ribozyme in so-called metal ion rescue experiments [17, 26, 31–33]. Nitrogen prefers to coordinate to comparatively soft metal ions (e.g., Mn^2+^ over Mg^2+^), as has been observed in prior work [19, 21]. If a G -OH group forms a metal ion interaction, then the simplest expectation would be that replacing that -OH with a -NH_2_ group would result in weak binding in Mg^2+^ alone and stronger binding upon addition of Mn^2+^. In contrast, the absence of this signature would provide no evidence for a metal ion / -OH group contact, but, as a negative result, would also not provide strong evidence against such an interaction.

We also measured binding of 2′- and 3′-deoxyguanosine (G(2′H) and G(3′H), respectively) to the *Azoarcus* ribozyme. The hydrogen (-H) substituent, unlike the -OH group, can neither coordinate with a metal ion nor form a hydrogen bond. Thus, disruption of G binding by -H substitution would suggest that a stabilizing interaction is made with the corresponding -OH group, although smaller effects can potentially arise from differential sugar pucker preferences.

**Fig 2:**
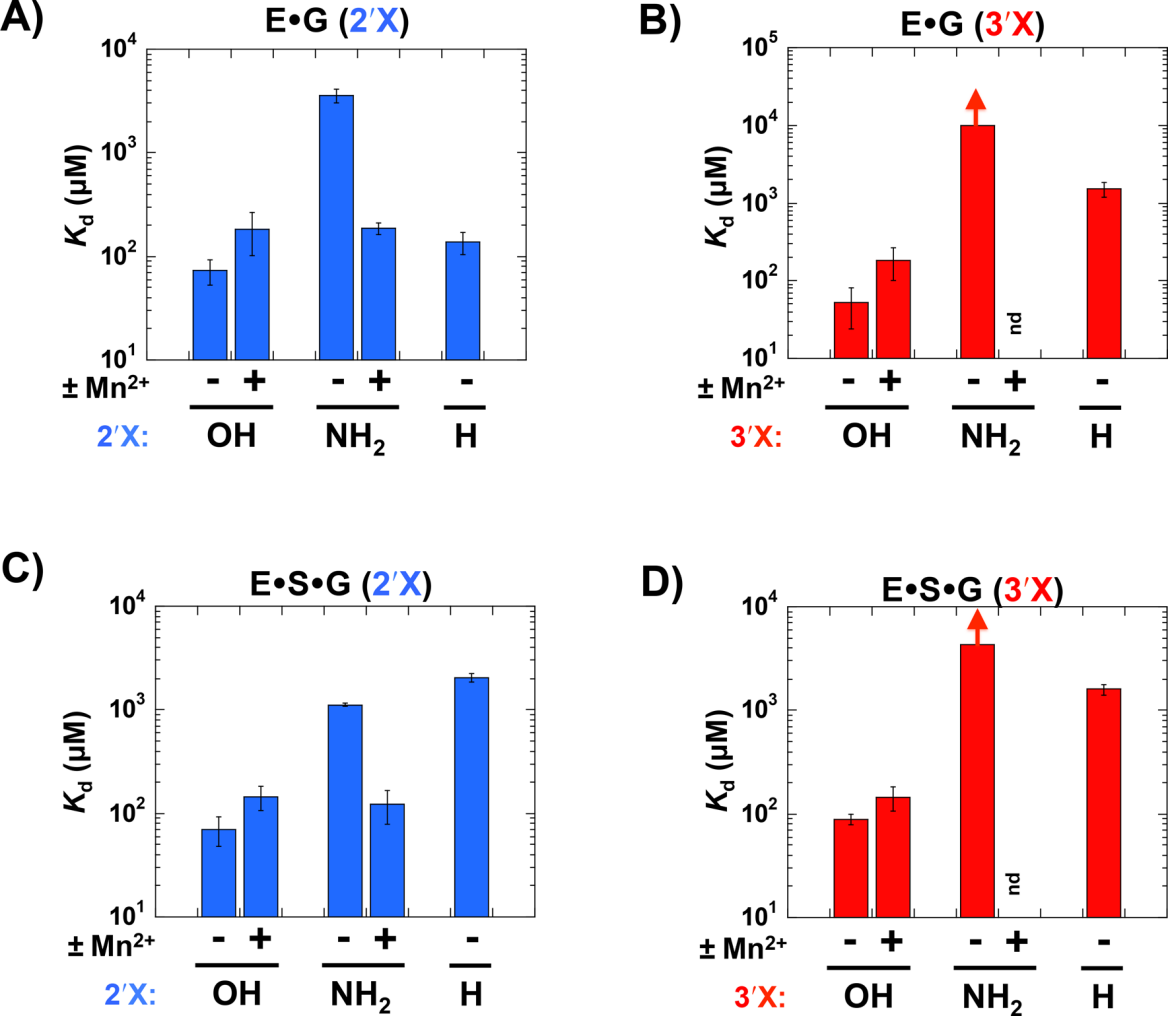
Effects of 2′- and 3′-modifications on G binding to the *Azoarcus* ribozyme. Equilibrium binding constants (*K*_d_s) were obtained as described in Materials and Methods. (A,C) The effects of 2′-NH_2_ and 2′-H substitutions on G binding to E (A) and ES (C). Values for binding of G represent the mean equilibrium binding constant from several independent measurements at different pH values at 15 mM Mg^2+^ in the absence or presence of 10 mM Mn^2+^ (S1 and S2 Figs). Values for binding of G(2′NH_2_) are derived from the fit to the model shown in S4–S6 Figs. The value for binding of G(2′NH_2_) to ES in the presence of Mn^2+^ represents the mean equilibrium binding constant from several independent measurements (S7 Fig). The fits to determine the *K*_d_ for G(2′H) are shown in S10 Fig. (B,D) The effects of 3′-NH_2_ and 3′-H substitutions on G binding to E (B) and ES (D). Values for binding of G at 15 mM Mg^2+^ were obtained as described above. Values for binding of G(3′NH_2_) were derived from the fit to the model shown in S8 and S9 Figs. Measurements were made at 100 mM Mg^2+^ to attenuate strong binding of G(3′N${\text{H}}_{3}^{+}$) (Materials and Methods). The arrows for the binding constants of G(3′NH_2_) denote that the observed *K*_d_s are lower limits (S8 and S9 Figs). Raising the Mg^2+^ concentration to 100 mM had a negligible effect on binding of G (S3 Fig), so that the affinities of G(3′NH_2_) can be compared with binding affinities for G at 15 mM Mg^2+^. The fits to determine the *K*_d_ for G(3′H) are shown in S10 Fig.

Equilibrium dissociation constants for binding of the G analogs to the *Azoarcus* E•S complex were measured following procedures described in Materials and Methods, and our results are summarized in Fig 2C and 2D. Replacing the 2′- or 3′-OH group with an -NH_2_ group has 17- or ≥66-fold destabilizing effects on G binding, respectively. The upper limit of ≥66 was set for G(3′NH_2_) binding due to the dominant contribution of the $\text{-}N{\text{H}}_{3}^{+}$ species to the observed affinity under experimentally accessible conditions (Materials and Methods, S9 Fig). Mn^2+^ enhances binding of G(2′NH_2_) to E•S so that its binding affinity is within error to that of G (Fig 2C). We could not determine Mn^2+^ rescue for G(3′NH_2_), due to the dominant binding of the $\text{-}N{\text{H}}_{3}^{+}$ species. The weakened binding of the G 2′- and 3′-H substitution, by 31-fold and 24-fold, respectively, provided additional evidence for interactions with both–OH groups.

The Mn^2+^ specificity switch observed for G(2′NH_2_) is consistent with preliminary rescue experiments [34] and crystallographic data [30] and, along with the destabilizing effect of 2′-H substitution, provides strong evidence for a direct metal ion interaction with the G 2′-OH group in the E•S•G complex. Given the observation that the 3′-OH group is within coordination distance of M_C_ in the *Azoarcus* crystal structure [30], the drop in affinity for G(3′H) and G(3′NH_2_) is most simply explained by the disruption of a stabilizing interaction with M_C_ and the G 3′-moiety. Nevertheless, we could not obtain direct evidence for this metal ion interaction or more strongly rule out alternative models involving disruption of other interactions because of technical limitations that precluded metal ion rescue experiments at this position (see above and Materials and Methods). The inferred metal ion interactions with the G 2′- and 3′-OH groups are equivalent to contacts made with the G -OH groups in the *Tetrahymena* E•S•G complex (Fig 1B) and suggest that both the *Tetrahymena* and *Azoarcus* ribozymes form the metal ion interactions required for catalysis in the presence of bound S.

We next probed for interactions made with the G -OH groups in the *Azoarcus* E•G complex. We measured binding of the G analogs under conditions in which S is not predominantly bound to the ribozyme (see Materials and Methods), and our results are summarized in Fig 2A and 2B. As observed above for E•S, replacing either the 2′- and 3′-OH group with an -NH_2_ group had strong destabilizing effects on binding of G, 51- and ≥143-fold, respectively (Fig 2A). In addition, Mn^2+^ restored binding of G(2′NH_2_) to levels similar to that for G binding (Fig 2A). Mn^2+^ rescue of G(3′NH_2_) binding could not be tested for the reasons described above. Finally, 3′-H substitution weakened binding by 22-fold (Fig 2B). Unexpectedly, no deleterious effect was observed for binding of G(2′H) to E, despite the evidence for a metal ion interaction with the 2′-OH group.

The Mn^2+^ specificity switch observed for G(2′NH_2_) binding provides strong evidence that the 2′-OH group contacts a metal ion in E•G. Although we were unable to probe for an analogous switch for G(3′NH_2_) binding due to technical limitations described above, the observation that the 3′-NH_2_ and 3′-H substitutions weaken G binding is indicative of a stabilizing interaction being made with the G 3′-OH group. Although more complex models are possible, analogous functional results were obtained for the E•S•G complex (Fig 2C and 2D), where there is also direct x-ray evidence for 2′- and 3′-OH interactions with M_C_ [30]. We therefore adopt the simplest model in which these same interactions are present in the *Azoarcus* E•G complex.

As for the absence of a deleterious effect for 2′H substitution to E, we postulate that the deoxyribose ring of the G(2′H) binds to the ribozyme in an alternative configuration in which the 2′-H atom faces away from the active site metal ions. This configuration would allow the metal ions to remain solvated and thereby avoid a substantial energetic penalty and allow its binding energy to coincidentally match that for G. Such enhanced flexibility may be facilitated by the small size of the–H atom and the alternative sugar conformation of the deoxyribose ring that would be restricted with the bulkier–NH_2_ substituent or when S is present in the active site.

For the *Tetrahymena* ribozyme, there is strong evidence for the absence of an interaction with the 2′-OH group of G and for the presence of a nonproductive contact between the adjacent 3′-OH group and M_A_ (Fig 1B, [19]). In contrast, the *Azoarcus* data suggest interactions with both the 2′- and 3′-OH groups (Fig 2). These results imply that the off-pathway configuration observed for the *Tetrahymena* ribozyme is not present for the *Azoarcus* ribozyme.

## Discussion

**Fig 3:**
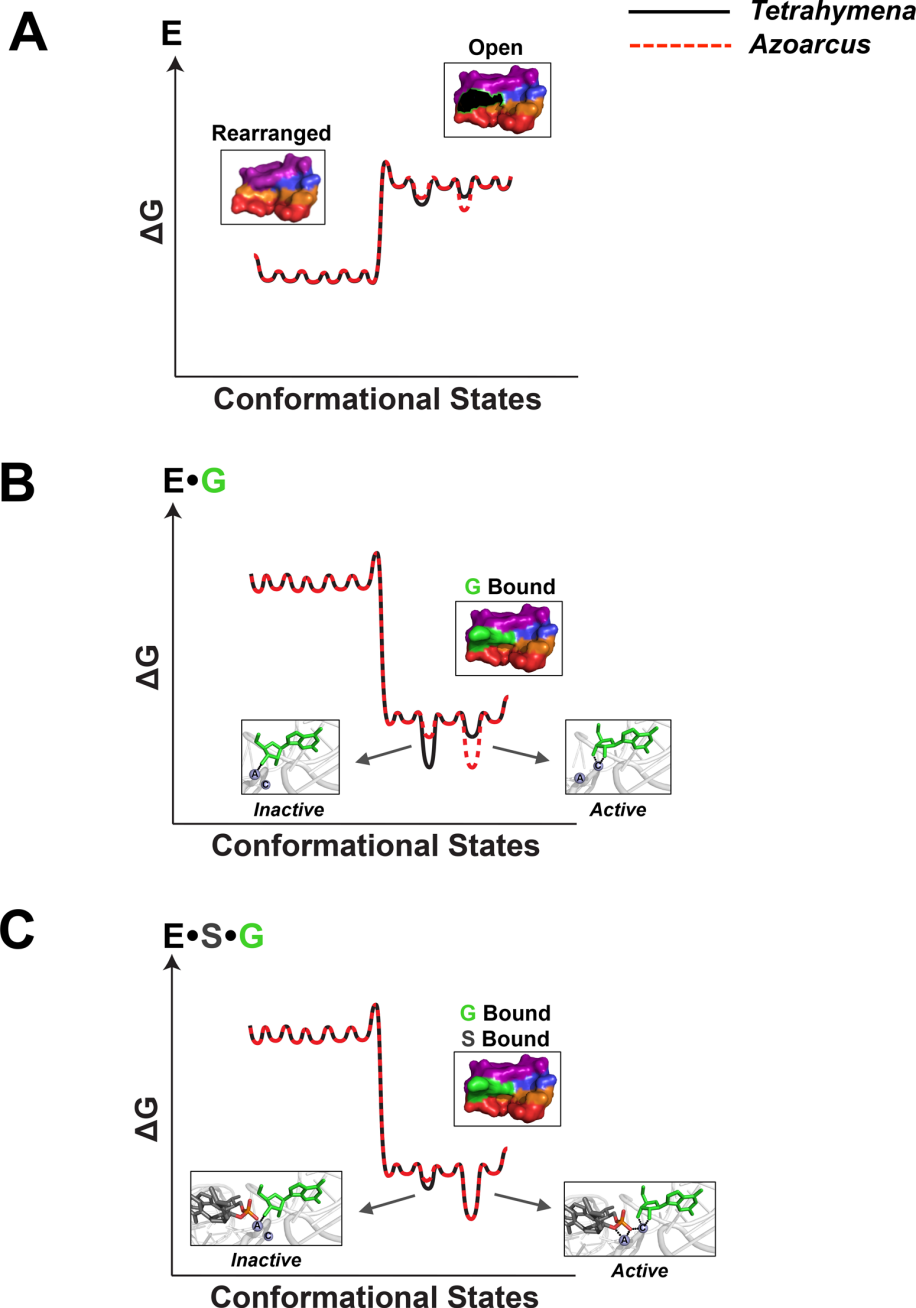
Conformational landscapes of the G binding site. Qualitative energy landscapes of the G binding sites of E (A) and the EG (B) and ESG complex (C), for the *Tetrahymena* and *Azoarcus* ribozymes (black and red lines, respectively). The G binding site is shown as a surface representation with or without bound G (green). G is also represented in stick form in panels B and C to highlight active site metal ion interactions in the inactive and active states [19]. The energy landscape and the accompanying structures were constructed as described in Materials and Methods.

We present a model for molecular recognition of G by the *Tetrahymena* and *Azoarcus* group I ribozymes (Fig 3), based on data described here and elsewhere [8, 19, 23]. The free form of the G binding site, as suggested from the very slow rate of G association to the *Azoarcus* and *Tetrahymena* ribozymes (~10^5^ M^-1^ min^-1^, [23]), favors one or more G-inaccessible (“rearranged,” Fig 3A) configurations for both ribozymes. This scenario is illustrated in the qualitative energy landscape for the G binding site of E of Fig 3A, which shows similar profiles for the *Azoarcus* and *Tetrahymena* ribozymes.

Transient formation of the G-accessible (“open”, Fig 3A) state allows G to bind to the ribozyme. However, our results suggest that the assembly of catalytic interactions with bound G is different for the *Tetrahymena* and *Azoarcus* ribozymes. Prior results with the *Tetrahymena* ribozyme [19] suggest that bound G is in equilibrium between an active and an inactive configuration in which the G -OH groups do and do not contact M_C_, respectively. The inactive form is preferred within E•G (Figs 1B and 3B) and the active form is favored once S is bound (E•S•G, Figs 1B and 3C).

In contrast, our results and prior x-ray structural results [30] with the *Azoarcus* ribozyme indicate interactions with both the G -OH groups and most simply suggest that the conformation in which both -OH groups coordinate to M_C_, is preferred for both E•G and E•S•G (Fig 3B and 3C, red). This model implies that the active site of the *Azoarcus* ribozyme does not significantly populate the alternative off-pathway state present for the *Tetrahymena* ribozyme and suggests an energy landscape for the *Azoarcus* ribozyme that is more constrained towards the active form than that for the *Tetrahymena* ribozyme. This difference occurs despite both introns having resting unbound states that are G-inaccessible (Fig 3A, “rearranged”) and highly similar G-occupied binding sites in the E•S•G complexes that overlay with an RMSD of <1 Å [35]. Phylogenetic, mechanistic, and interference studies suggest that the active sites of the *Tetrahymena* and *Azoarcus* ribozymes are highly similar [23, 34, 36, 37] and thus favor models in which local rather than global rearrangements occur to assemble the catalytically active conformation.

The ability to access local conformational differences to assemble catalytic interactions suggests a degree of flexibility within the group I ribozyme active site. This flexibility is consistent with metal ion rescue experiments for the *Tetrahymena* ribozyme that implicate a third metal ion, M_B_, that contacts the G 3′-oxygen atom instead of M_C_ in the transition state (Fig 1B, [25]). Functional studies with the *Tetrahymena* ribozyme provided strong evidence for a transition state interaction of the G 3′-atom with a weakly associating metal ion (i.e. M_B_) that is distinct from M_A_ and M_C_, but M_B_ has not been observed in any of the group I intron x-ray structures [25, 30, 35]. These functional studies employed metal ion rescue of 3′-thio-substituted G [25]. However, recent results suggest that for metal ion rescue of 3′-amino-substituted G, M_C_ instead of M_B_ interacts with the 3′-atom in the E•S•G ground state complex [19].

The simplest model to account for these seemingly disparate results is that with the G 3′-sulfur the active site of the *Tetrahymena* ribozyme is sufficiently malleable to recruit M_B_ to form a non-cognate contact. Thus, it appears that the group I active site, while likely utilizing two metal ions for its cognate reaction, can readily toggle between a two metal ion and three metal ion mechanism for catalysis. These observations appear to be a manifestation of RNA’s ability to adopt multiple distinct conformational–and functional–states [12, 38–41].

While the rate of G association to the *Tetrahymena* and *Azoarcus* ribozymes is the same [23], G association to the *Twort* group I ribozymes is ~10-fold faster than to the *Tetrahymena* and *Azoarcus*. This, together with weaker binding of G to the *Anabaena* ribozyme relative to *Azoarcus*, suggests a range in G association rate constants [23, 42, 43]. It will be fascinating to uncover the structural variation, whether local or global, that differentially impacts these conserved cores.

Our results highlight RNA’s ability and tendency to access local conformational differences, even within the conserved catalytic core of a ribozyme. This feature of RNA is common (e.g. [11, 26, 40, 44–48]), and may have been a valuable asset for Nature in its evolution of complex RNA machines such as the ribosome and splicosome that must access multiple conformational states [12–14, 39, 49]. It will thus be of substantial interest to elucidate the aspects of RNA structure that dictate its ability to restrict or access multiple conformational states, and model systems such as group I ribozymes may provide valuable clues and tools.

## Materials and Methods

### Materials

The L-6 *Azoarcus* ribozyme (E) was prepared as described previously [23]. RNA oligonucleotides were purchased from Dharmacon Inc. (Lafayette, CO), 5′-^32^P-radiolabeled using [γ-^32^P]ATP (MP Biomedicals, Santa Ana, CA) and T4 polynucleotide kinase (New England Biolabs, Ipswich, MA) according to the manufacturer’s protocol, and gel purified following standard procedures [50]. 2′-deoxyguanosine (G(2′H)) and 3′-deoxyguanosine (G(3′H)) were purchased from Sigma-Aldrich (Saint Louis, MO) and 2′- and 3′-aminoguanosine (G(2′N) and G(3′N), respectively) were purchased from Santa Cruz Biotechnology (Santa Cruz, CA). The amino group of G(2′N) and G(3′N) exists in neutral -NH_2_ and protonated -N${\text{H}}_{3}^{+}$ forms. We refer to the -NH_2_ form as G(2′NH_2_) and G(3′NH_2_), respectively and the -N${\text{H}}_{3}^{+}$ forms as G(2′N${\text{H}}_{3}^{+}$) and G(3′N${\text{H}}_{3}^{+}$), respectively.

In experiments with G(2′H) and G(3′H), the deoxyguanosine analogs were pretreated with 26 mM sodium periodate to convert any guanosine contaminant to an unreactive dialdehyde, as described previously [19]. After 1 h incubation at room temperature in the dark, the remaining sodium periodate was quenched by adding excess ethylene glycol and incubating for another hour. Stocks of G(2′H) and G(3′H) were subsequently diluted at desired concentrations. This procedure did not affect ribozyme-mediated cleavage activity [19].

### General kinetic methods

All cleavage reactions were single-turnover with ribozyme in excess of trace ^32^P-radiolabeled substrate (< 0.1 nM), and were carried out at 30°C in the presence of 15 mM MgCl_2_ [23] and 50 mM buffer at various pH values (sodium acetate, pH 5.0–5.5; NaMES, pH 6.1–6.7; NaMOPS, pH 7.1; NaEPPS, pH 7.7–8.2; and NaCHES, pH 8.7–9.7). For metal ion rescue experiments, reactions were performed in the absence or presence of 10 mM MnCl_2_. For G(3′N), reactions were performed in the presence of 100 mM Mg^2+^ to attenuate strong binding of the 3′-N${\text{H}}_{3}^{+}$ form of the analog (S8 and S9 Figs).

Ribozymes were allowed to fold for 30 min at 50°C in 15 mM MgCl_2_ and 50 mM buffer at the pH of the final reaction mixture. For reactions performed above pH 8.0, the ribozyme was folded in 25 mM NaMES, pH 6.7, and 15 mM MgCl_2_ to minimize degradation of the ribozyme. After cooling to room temperature, the mixture was diluted 10-fold in a solution containing the desired concentration of buffer, G analog, and divalent metal ions. All reactions were allowed to equilibrate for 5–10 min at 30°C before initiating the cleavage reaction by the addition of ^32^P-radiolabeled substrate. At specific times, 2 μL aliquots were taken from the 20 μL reaction mixture and quenched in 4 μL quench solution containing 90% formamide, 50 mM EDTA, bromophenol blue and xylene cyanole. ^32^P-radiolabeled substrate and product were separated by gel electrophoresis on a denaturing polyacylamide gel (7M urea/20%acrylamide) and quantitated using Phosphorimager analysis (GE Healthcare) with TotalLab.

Reactions were followed to completion and generally exhibited endpoints >95%. Slow reactions were followed for up to 24 hours and rate constants were obtained from initial rates assuming 95% endpoints. Data were analyzed using KaleidaGraph, Synergy Software and errors reported represent the standard error values of the parameters obtained by fitting, unless stated otherwise.

### Measuring the affinity of G to E and E•S

Equilibrium dissociation constants for G from E•G and E•G•S ($K_{\text{d}}^{\text{G}}$ and KdG', respectively, Fig 1A) were determined by plotting the observed rate (*k*_obs_) of cleavage of 5′-^32^P-radiolabeled CAUA_5_ (or CAdUA_5_) as a function of the concentration of G (0–2.5 mM G), and fitting the data to Eq 1:

$$k_{\text{obs}}=\frac{k_{\text{max}}\;[\text{G}]}{[\text{G}]+K_{1/2}^{\text{G}}}$$ (1)

$K_{1/2}^{\text{G}}$, the concentration of G where the observed rate constant (*k*_obs_) is half the maximal rate (*k*_max_), is equal to $K_{\text{d}}^{\text{G}}$ (or KdG') when the chemical step is rate-limiting. For the CAUA_5_ substrate, the chemical step is rate-limiting at pH <6.3 [23]. Above this pH, we used the CAdUA_5_ substrate in which the cleavage site 2′-OH group is replaced with a hydrogen atom. This modification slows the reaction rate by ~1000-fold so that chemistry is rate limiting at higher pH [23, 51].

Binding of G to E•S was monitored under conditions where E is saturating with respect to S ([E] = 500 nM, $K_{\text{d}}^{\text{S}}$ = 68 nM) [23]. To monitor binding of G to E, a subsaturating enzyme concentration (5–20 nM E) was used. Binding constants of G determined in this work are within ~2-3-fold with previously published affinities [23]. Since binding of G is pH independent over the range measured (S1–S3 Figs), affinities for G are represented as an average of independent measurements at various pH with the error bars representing the corresponding standard deviation.

### Measuring the affinities of G(2′H), G(3′H), and G(3′N) to E and E•S

G(2′H), G(3′H), and G(3′N) are essentially non-reactive [19] so binding of these analogs to E and E•S was measured via competitive inhibition of the reaction with G. At low pH (< pH 7.0), we used the CAUA_5_ substrate to accelerate the rate of reaction. Experiments were carried out with subsaturating G (10–30 μM G) and with varying concentrations of G(2′H), G(3′H), and G(3′N) (0–3 mM) at the appropriate [E] to ensure that binding to E or E•S was being monitored (see above). For each G analog (G_X_), the data were fit to Eq 2:

$$k_{\text{obs}}=\frac{k_{\text{max}}\;K_{\text{i}}}{[\text{G}]+K_{\text{i}}}$$ (2)

*K*_i_, the inhibitory concentration at which the observed rate constant (*k*_obs_) is half the maximal rate (*k*_max_), is equal to the equilibrium dissociation constant ($K_{\text{d}}^{{\text{G}}_{\text{x}}}$ or KdGx').

### Measuring the affinity of G(2′N) to E and E•S

The affinity of G(2′N) to E and E•S was measured using an activity assay following procedures analogous to what we described for G (see above). Equilibrium dissociation constants for G(2′N) were determined by plotting the observed rate (*k*_obs_) of cleavage of 5′-^32^P-radiolabeled CAdUA_5_ as a function of the concentration of G(2′N) (0–2.5 mM), and fitting the data to Eq 3.

$$k_{\text{obs}}=\frac{k_{\text{max}}\;[\text{G}(2'\text{N})]}{{[\text{G}(2'\text{N})]+K}_{1/2}^{\text{G}(2'\text{N})}}$$ (3)

### Following Binding of the–NH_2_ and–N${\text{H}}_{3}^{+}$ forms of G(2′N) and G(3′N)

The -NH_2_ group on G(2′N) and G(3′N) ionizes with p*K*_a_ values of 6.2 and 7.0, respectively [52, 53], to form the corresponding -N${\text{H}}_{3}^{+}$ species. To ensure that we were monitoring binding of the -NH_2_ and not the -N${\text{H}}_{3}^{+}$ species [19, 54], we determined the pH-dependence of G(2′N) and G(3′N) binding to E and E•S. The data were fit to a model for binding of the -NH_2_ and—N${\text{H}}_{3}^{+}$ forms of G(2′N) and G(3′N), as summarized in our Supplementary Figures (S4–S6, S8 and S9 Figs). Our data indicate that G(2′N${\text{H}}_{3}^{+}$) and G(3′N${\text{H}}_{3}^{+}$) bind strongly to the *Azoarcus* ribozyme (S8-S9), analogous to observations made with the *Tetrahymena* ribozyme [19, 54]. Strong binding presumably arises from favorable electrostatic interactions with the -N${\text{H}}_{3}^{+}$ species in the active site of these ribozymes. The high affinity of G(3′N${\text{H}}_{3}^{+}$) obscured our measurements of G(3′NH_2_) at the highest accessible pH value without significant ribozyme degradation in the presence of Mn^2+^ (pH 7.7). Metal ion rescue of G(3′NH_2_) binding could thus not be tested.

### Construction of qualitative energy landscapes for the *Azoarcus* and *Tetrahymena* G binding sites

Here, we describe the rationale behind the conformational landscapes depicted in Fig 3. Within E, prior data suggests that the “open” or G-accessible state is not the most stable species at equilibrium and that the preferred state may correspond to one or more collapsed or rearranged configurations that prevent binding of G [8, 23]. The surface representation of the open state was obtained by removing G from the G binding site of the *Tetrahymena* group I intron (PDB 1X8W), and the rearranged state was built from the structure of the *Tetrahymena* group I intron using Assemble to alter the position of the top base triple so that G binding is occluded [18]. We emphasize that these structures are models of possible states in the conformational ensemble of the G binding site (see [19] for additional discussion). Binding of G stabilizes the G accessible state (as depicted by the change in relative energies of the rearranged and open states) and, within E•G, bound G can exist in (at least) two configurations referred to as “active” and “inactive”. Prior data [19] suggest that the inactive state is the preferred state in *Tetrahymena*, whereas the work described herein suggests that the active state is preferred in *Azoarcus*. To represent this, the well for the active state is lower in energy than the well for the inactive state. Within E•S•G, the active site of the *Tetrahymena* ribozyme undergoes a rearrangement to the active state, which is preferred for both the *Azoarcus* and *Tetrahymena* ribozymes.

## Supporting Information

S1 FigBinding of G to E and E•S in a background of 15 mM Mg^2+^.(A,B) G concentration dependence of the normalized rate of cleavage ($k_{\text{obs}}^{\text{norm}}$) for E (A) and E•S (B). Measurements were made in a 15 mM Mg^2+^ background at pH 7.7 (purple), 8.8 (grey), and 9.2 (green).The lines are fits of the data from Eq 1 in Materials and Methods. (C,D) pH-dependence of G binding ($K_{\text{a},\text{obs}}^{\text{G}}$ = 1/$K_{\text{d},\text{obs}}^{\text{G}}$) to E (C) and E•S (D) from the data shown in (A) and (B), respectively. The lines correspond to the mean of the measured G binding affinities to E (14.3 ± 4 mM^-1^) and E•S (15.2 ± 1 mM^-1^). Binding affinities are summarized in the S1 File.(TIF)

S2 FigBinding of G to E and E•S in the presence of Mn^2+^.(A,B) G concentration dependence of the normalized rate of cleavage ($k_{\text{obs}}^{\text{norm}}$) for E (A) and E•S (B). Measurements were made in the presence of 15 mM Mg^2+^ and 10 mM Mn^2+^ at pH 6.7 (pink), 7.1 (bright green), and 7.7 (purple). The lines are fits of the data from Eq 1 in Materials and Methods. (C,D) pH-dependence of G binding ($K_{\text{a},\text{obs}}^{\text{G}}$ = 1/$K_{\text{d},\text{obs}}^{\text{G}}$) to E (C) and E•S (D) from the data shown in (A) and (B), respectively. The lines correspond to the mean of the measured G binding affinities to E (6.6 ± 4 mM^-1^) and E•S (7.3 ± 2 mM^-1^). Binding affinities are summarized in the S1 File.(TIF)

S3 FigBinding of G to E and E•S in a background of 100 mM Mg^2+^.(A,B) G concentration dependence of the normalized rate of cleavage ($k_{\text{obs}}^{\text{norm}}$) for E and E•S (B) in the presence of 100 mM Mg^2+^. Measurements were made at pH 5.5 (orange), 6.1 (blue), 7.1 (bright green), 7.7 (purple), 8.2 (bright blue), 8.8 (grey), and 9.2 (green). The different symbols (circles and triangles) at pH 7.7 and 9.2 denote two independent measurements made at these pH values. The line is a fit of the data from Eq 1 in Materials and Methods. (C,D) pH-dependence of G binding ($K_{\text{a},\text{obs}}^{\text{G}}$ = 1/$K_{\text{d},\text{obs}}^{\text{G}}$) to E (C) and E•S (D) from the data shown in (A) and (B), respectively. The dashed lines are the mean of the measured affinities of G to E (21.9 ± 18 mM^-1^) and E•S (10.1 ± 3 mM^-1^). Binding affinities are summarized in the S1 File.(TIF)

S4 FigBinding of G(2′N) to E.G(2′N) concentration dependence of the normalized rate of cleavage ($k_{\text{obs}}^{\text{norm}}$) for E. Measurements were made at pH 7.7 (purple), 8.2 (bright blue), 8.4 (red), 8.8 (grey), and 9.2 (green). The lines are fits of the data from Eq 3 in Materials and Methods. (B) pH-dependence of G(2′N) (black) and G (grey) binding to E. The binding affinities ($K_{\text{a},\text{obs}}^{\text{Gx}}$ (= 1/$K_{\text{d},\text{obs}}^{\text{Gx}}$) for G(2′N) and G were obtained from the data in (A) and S1A Fig, respectively. The solid line (black) is a fit of the G(2′N) data according to the model shown in (C) which describes binding of the -NH_2_ and -N${\text{H}}_{3}^{+}$ forms of G(2′N) to E. For comparison, the data was also fit to a model in which the–NH_2_ form of G(2′N) does not bind to E (dotted line). The solid line (grey) is the average of the measured G affinities to E (14.3 mM^-1^; see S1C Fig). (C) Model for binding of G(2′NH_2_) and G(2′N${\text{H}}_{3}^{+}$) to E. $K_{\text{a}}^{\text{G}(2'\text{N}{\text{H}}_{2})}$ and $K_{\text{a}}^{\text{G}(2'\text{N}{\text{H}}_{3}^{+})}$ report binding of G(2′NH_2_) and G(2′N${\text{H}}_{3}^{+}$) to E, respectively. A limit of >5 mM^-1^ was set for $K_{\text{a}}^{\text{G}(2'\text{N}{\text{H}}_{3}^{+})}$ since binding of G(2′N) did not level off at pH 7.7 (($K_{\text{a},\text{obs}}^{\text{G}(2'\text{N})}$ = 5 mM^-1^). p$K_{\text{a}}^{\text{G}(2'\text{N})}$ and p$K_{\text{a}}^{\text{E}•\text{G}(2'\text{N})}$ are the equilibrium constants for deprotonation of G(2′N${\text{H}}_{3}^{+}$) in solution and in E•G(2′N), respectively. The value of p$K_{\text{a}}^{\text{G}(2'\text{N})}$ was set to 6.2 [52–54], and p$K_{\text{a}}^{\text{E}•\text{G}(2'\text{N})}$ was determined by completing the thermodynamic cycle. Binding affinities are summarized in the S1 File.(TIF)

S5 FigBinding of G(2′N) to E in the presence of Mn^2+^.(A) G(2′N) concentration dependence of the normalized rate of cleavage ($k_{\text{obs}}^{\text{norm}}$) for E in the presence of 15 mM Mg^2+^ and 10 mM Mn^2+^. Measurements were made at pH 6.7 (pink), 7.1 (bright green), and 7.7 (purple). The different symbols (circles and triangles) at pH 7.7 denote two independent measurements made at this pH. The lines are fits of the data from Eq 3 in Materials and Methods. (B) pH-dependence of G(2′N) (black) and G (grey) binding to E. The binding affinities ($K_{\text{a},\text{obs}}^{\text{Gx}}$ (= 1/$K_{\text{d},\text{obs}}^{\text{Gx}}$) for G(2′N) and G were obtained from the data in (A) and S2A Fig, respectively. The black line is a fit of the G(2′N) data according to the model shown in (C) that describes binding of the -NH_2_ and -N${\text{H}}_{3}^{+}$ forms of G(2′N) to E. The grey line is the average of the measured G affinities to E (6.6 mM^-1^; see S1C Fig). (C) Model for binding of G(2′NH_2_) and G(2′N${\text{H}}_{3}^{+}$) to E. $K_{\text{a}}^{\text{G}(2'\text{N}{\text{H}}_{2})}$ and $K_{\text{a}}^{\text{G}(2'\text{N}{\text{H}}_{3}^{+})}$ report binding of G(2′NH_2_) and G(2′N${\text{H}}_{3}^{+}$) to E. A limit was set for $K_{\text{a}}^{\text{G}(2'\text{N}{\text{H}}_{3}^{+})}$ because binding of G(2′N) does not level off at the lowest pH measured. p$K_{\text{a}}^{\text{G}(2'\text{N})}$ and p$K_{\text{a}}^{\text{E}•\text{G}(2'\text{N})}$ are the equilibrium constants for deprotonation of G(2′N${\text{H}}_{3}^{+}$) in solution and in E•G(2′N), respectively. p$K_{\text{a}}^{\text{G}(2'\text{N})}$ was set to 6.2 [52–54], and p$K_{\text{a}}^{\text{E}•\text{G}(2'\text{N})}$ was determined by completing the thermodynamic cycle. Binding affinities are summarized in the S1 File.(TIF)

S6 FigBinding of G(2′N) to E•S.(A) G(2′N) concentration dependence of the normalized rate of cleavage ($k_{\text{obs}}^{\text{norm}}$) for E•S in the presence of 15 mM Mg^2+^. Measurements were made at pH 7.7 (purple), 8.4 (red), pH 8.8 (grey), and pH 9.2 (green). The different symbols (circles and triangles) at pH 7.7 and 8.8 denote two independent measurements made at these pH values. The lines are fits of the data from Eq 3 in Materials and Methods. (C) pH-dependence of G(2′N) (black) and G (grey) binding to E•S. The binding affinities ($K_{\text{a},\text{obs}}^{\text{Gx}}$ (= 1/$K_{\text{d},\text{obs}}^{\text{Gx}}$) for G(2′N) and G were obtained from the data in (A,B) and S1B Fig, respectively. The black line is a fit of the G(2′N) data according to the model shown in (C) which describes binding of the -NH_2_ and -N${\text{H}}_{3}^{+}$ forms of G(2′N) to E•S. The grey line is the average of the measured G affinities to E•S (15.2 mM^-1^; see S1D Fig). (C) Model for binding of G(2′NH_2_) and G(2′N${\text{H}}_{3}^{+}$) to E•S. $K_{\text{a}}^{\text{G}(2'\text{N}{\text{H}}_{2})}$ and $K_{\text{a}}^{\text{G}(2'\text{N}{\text{H}}_{3}^{+})}$ report binding of G(2′NH_2_) and G(2′N${\text{H}}_{3}^{+}$) to E•S, respectively. A limit of >5 mM^-1^ was set for $K_{\text{a}}^{\text{G}(2'\text{N}{\text{H}}_{3}^{+})}$ as binding of G(2′N) did not level off at pH 7.7 ($K_{\text{a},\text{obs}}^{\text{G}(2'\text{N})}$ = 5 mM^-1^). p$K_{\text{a}}^{\text{G}(2'\text{N})}$ and p$K_{\text{a}}^{\text{E}•\text{S}•\text{G}(2'\text{N})}$ are the equilibrium constants for deprotonation of G(2′N${\text{H}}_{3}^{+}$) in solution and in E•S•G(2′N), respectively. p$K_{\text{a}}^{\text{G}(2'\text{N})}$ is 6.2 [52–54], and p$K_{\text{a}}^{\text{E}•\text{S}•\text{G}(2'\text{N})}$ was determined by completing the thermodynamic cycle. Binding affinities are summarized in the S1 File.(TIF)

S7 FigBinding of G(2′N) to E•S in the presence of Mn^2+^.(A) G(2′N) concentration dependence of the normalized rate of cleavage ($k_{\text{obs}}^{\text{norm}}$) for E•S in the presence of 15 mM Mg^2+^ and 10 mM Mn^2+^. Measurements were made at pH 7.1 (bright green) and pH 7.7 (purple). The different symbols (circles and triangles) at pH 7.1 and 7.7 denote two independent measurements made at these pH values. The lines are fits of the data from Eq 3 in Materials and Methods. (B) pH-dependence of G(2′N) (black) and G (grey) binding to E•S. The binding affinities ($K_{\text{a},\text{obs}}^{\text{Gx}}$ (= 1/$K_{\text{d},\text{obs}}^{\text{Gx}}$) for G(2′N) and G were obtained from the data in (A) and S2B Fig, respectively. The lines are the average of the measured G (grey line, 7.3 mM^-1^; S2D Fig) and G(2′N) (black line, 8.1 ± 3 mM^-1^) affinities to E•S. The observation that varying the pH from 7.1 to 7.7 does not significant change $K_{\text{a},\text{obs}}^{\text{G}(2'\text{N})}$ suggests that the 2′-amino group exists in the -NH_2_ form above pH 7.1 [52–54].(TIF)

S8 FigBinding of G(3′N) binding to E.(A) G(3′N) inhibition of the normalized rate of cleavage ($k_{\text{obs}}^{\text{norm}}$) of G with E and S in the presence of 100 mM Mg^2+^. Measurements were made at pH 5.5 (black), 6.5 (pink), 7.7 (purple), 8.4 (red), 8.8 (grey), and 9.2 (green). The different symbols (circles and triangles) at pH 9.2 denote two independent measurements made at this pH. The lines are fits of the data from Eq 2 in Materials and Methods. (B) pH-dependence of G(3′N) (black) and G (grey) binding to E. The binding affinities ($K_{\text{a},\text{obs}}^{\text{Gx}}$ (= 1/$K_{\text{d},\text{obs}}^{\text{Gx}}$) for G(3′N) and G were obtained from the data in (A) and S3A Fig, respectively. The solid line (black) is a fit of the G(3′N) data according to the model shown in (C) which describes binding of the -NH_2_ and -N${\text{H}}_{3}^{+}$ forms of G(3′N) to E. For comparison, the data was also fit to a model in which the–NH_2_ form of (3′N) does not bind to E (dotted line). The solid line (grey) is the average of the measured G affinities to E (21.9 mM^-1^; see S1C Fig). (C) Model for binding of G(3′NH_2_) and G(3′N${\text{H}}_{3}^{+}$) to E. $K_{\text{a}}^{\text{G}(3'\text{N}{\text{H}}_{2})}$ and $K_{\text{a}}^{\text{G}(3'\text{N}{\text{H}}_{3}^{+})}$ report binding of G(3′NH_2_) and G(3′N${\text{H}}_{3}^{+}$) to E, respectively, and were obtained from the fit of the data in (B). The $K_{\text{a}}^{\text{G}(3'\text{N}{\text{H}}_{2})}$ value is reported as a limit because binding of G(3′N) does not level off at high pH. p$K_{\text{a}}^{\text{G}(3'\text{N})}$ and p$K_{\text{a}}^{\text{E}•\text{G}(3'\text{N})}$ are the equilibrium constants for deprotonation of G(3′N${\text{H}}_{3}^{+}$) in solution and in E•G(3′N), respectively. p$K_{\text{a}}^{\text{G}(3'\text{N})}$ is 7.0 [52], and p$K_{\text{a}}^{\text{E}•\text{G}(3'\text{N})}$ was determined by completing the thermodynamic cycle. Binding affinities are summarized in the S1 File.(TIF)

S9 FigBinding of G(3′N) to E•S.(A) G(3′N) inhibition of the normalized rate of cleavage ($k_{\text{obs}}^{\text{norm}}$) of G with E•S in the presence of 100 mM Mg^2+^. Measurements were made at pH 5.5 (orange), 6.5 (pink), 7.7 (purple), 8.8 (grey), and 9.2 (green). The lines are fits of the data from Eq 2 in Materials and Methods. (B) pH-dependence of G(3′N) (black) and G (grey) binding to E•S. The binding affinities ($K_{\text{a},\text{obs}}^{\text{Gx}}$ (= 1/$K_{\text{d},\text{obs}}^{\text{Gx}}$) for G(3′N) and G were obtained from the data in (A) and S3B Fig, respectively. The solid line (black) is a fit of the G(3′N) data according to the model shown in (C) which describes binding of the -NH_2_ and -N${\text{H}}_{3}^{+}$ forms of G(3′N) to E•S. For comparison, the data was also fit to a model in which the–NH_2_ form of (3′N) does not bind to E•S (dotted line). The solid grey line is the average of the measured G affinities to E•S (10.1 mM^-1^; see S1D Fig). (C) Model for binding of G(3′NH_2_) and G(3′N${\text{H}}_{3}^{+}$) to E•S. $K_{\text{a}}^{\text{G}(3'\text{N}{\text{H}}_{2})}$ and $K_{\text{a}}^{\text{G}(3'\text{N}{\text{H}}_{3}^{+})}$ report binding of G(3′NH_2_) and G(3′N${\text{H}}_{3}^{+}$) to E•S, respectively, and were obtained from the fit of the data in (B). The $K_{\text{a}}^{\text{G}(3'\text{N}{\text{H}}_{2})}$ value is reported as a limit because binding of G(3′N) does not level off at high pH. p$K_{\text{a}}^{\text{G}(3'\text{N})}$ and p$K_{\text{a}}^{\text{E}•\text{S}•\text{G}(3'\text{N})}$ are the equilibrium constants for deprotonation of G(3′N${\text{H}}_{3}^{+}$) in solution and in E•S•G(3′N), respectively. p$K_{\text{a}}^{\text{G}(3'\text{N})}$ is 7.0 [52], and p$K_{\text{a}}^{\text{E}•\text{S}•\text{G}(3'\text{N})}$ was determined by completing the thermodynamic cycle. Binding affinities are summarized in the S1 File.(TIF)

S10 Fig Binding of G(2′H) and G(3′H) to E and E•S. (A) G(2′H) (black) and G(3′H) (red) inhibition of the reaction of G with E and S (A) or with E•S (B). Measurements were made at pH 7.0 in the presence of 15 mM Mg^2+^. The data were analyzed as described in Materials and Methods. Binding affinities are summarized in the S1 File.
(TIF)

S1 File Binding affinities used in S1–S10 Figs are compiled in the S1 File. (XLSX)

## References

[1] Williamson JR. Induced fit in RNA-protein recognition. Nat Struct Biol. 2000;7: 834–7. 11017187 10.1038/79575

[2] Hermann T, Patel DJ. Adaptive recognition by nucleic acid aptamers. Science. 2000;287: 820–5. 10657289 10.1126/science.287.5454.820

[3] Fedor MJ, Williamson JR. The catalytic diversity of RNAs. Nat Rev Mol Cell Biol. 2005;6: 399–412. 15956979 10.1038/nrm1647

[4] Serganov A, Patel DJ. Metabolite recognition principles and molecular mechanisms underlying riboswitch function. Annu Rev Biophys. 2012;41: 343–70. 10.1146/annurev-biophys-101211-113224 22577823 PMC4696762

[5] Sigler PB. An analysis of the structure of tRNA. Annu Rev Biophys Bioeng. 1975;4: 477–527. 1098566 10.1146/annurev.bb.04.060175.002401

[6] Herschlag D. RNA chaperones and the RNA folding problem. J Biol Chem. 1995;270: 20871–4. 7545662 10.1074/jbc.270.36.20871

[7] Liberman JA, Wedekind JE. Riboswitch structure in the ligand-free state. Wiley Interdiscip Rev RNA. 2012;3: 369–84. 10.1002/wrna.114 21957061 PMC3252462

[8] Karbstein K, Herschlag D. Extraordinarily slow binding of guanosine to the Tetrahymena group I ribozyme: implications for RNA preorganization and function. Proc Natl Acad Sci U S A. 2003;100: 2300–5. 12591943 10.1073/pnas.252749799 PMC151335

[9] Huang H, Suslov NB, Li NS, Shelke SA, Evans ME, Koldobskaya Y, et al A G-quadruplex-containing RNA activates fluorescence in a GFP-like fluorophore. Nat Chem Biol. 2014;10: 686–91. 10.1038/nchembio.1561 24952597 PMC4104137

[10] Ditzler MA, Rueda D, Mo J, Hakansson K, Walter NG. A rugged free energy landscape separates multiple functional RNA folds throughout denaturation. Nucleic Acids Res. 2008;36: 7088–99. 10.1093/nar/gkn871 18988629 PMC2602785

[11] Schmeing TM, Huang KS, Strobel SA, Steitz TA. An induced-fit mechanism to promote peptide bond formation and exclude hydrolysis of peptidyl-tRNA. Nature. 2005;438: 520–4. 16306996 10.1038/nature04152

[12] Voorhees RM, Ramakrishnan V. Structural basis of the translational elongation cycle. Annu Rev Biochem. 2013;82: 203–36. 10.1146/annurev-biochem-113009-092313 23746255

[13] Staley JP, Guthrie C. Mechanical devices of the spliceosome: motors, clocks, springs, and things. Cell. 1998;92: 315–26. 9476892 10.1016/s0092-8674(00)80925-3

[14] Guo Z, Karunatilaka KS, Rueda D. Single-molecule analysis of protein-free U2-U6 snRNAs. Nat Struct Mol Biol. 2009;16: 1154–9. 10.1038/nsmb.1672 19881500 PMC2784090

[15] Wickiser JK, Cheah MT, Breaker RR, Crothers DM. The kinetics of ligand binding by an adenine-sensing riboswitch. Biochemistry. 2005;44: 13404–14. 16201765 10.1021/bi051008u

[16] Ottink OM, Rampersad SM, Tessari M, Zaman GJ, Heus HA, Wijmenga SS. Ligand-induced folding of the guanine-sensing riboswitch is controlled by a combined predetermined induced fit mechanism. RNA. 2007;13: 2202–12. 17959930 10.1261/rna.635307 PMC2080608

[17] Hougland JL, Piccirilli JA, Forconi M, Lee J, Herschlag D. How the Group I Intron Works: A Case Study of RNA Structure and Function RNA World, Third Edition Cold Spring Harbor Monograph Series 43 Cold Spring Harbor: Cold Spring Harbor Laboratory Press; 2006 p. 133–205.

[18] Benz-Moy TL, Herschag D. Structure-function analysis from the outside in: Long-range tertiary contacts in RNA exhibit distinct catalytic roles. Biochemistry. 2011;50: 8733–55. 10.1021/bi2008245 21815635 PMC3186870

[19] Sengupta RN, Van Schie SN, Giambasu G, Dai Q, Yesselman JD, York D, et al An active site rearrangement within the Tetrahymena group I ribozyme releases nonproductive interactions and allows formation of catalytic interactions. RNA. 2016;22: 32–48. 10.1261/rna.053710.115 26567314 PMC4691833

[20] McConnell TS, Cech TR, Herschlag D. Guanosine binding to the Tetrahymena ribozyme: thermodynamic coupling with oligonucleotide binding. Proc Natl Acad Sci U S A. 1993;90: 8362–6. 8378306 10.1073/pnas.90.18.8362 PMC47356

[21] Shan SO, Herschlag D. Probing the role of metal ions in RNA catalysis: kinetic and thermodynamic characterization of a metal ion interaction with the 2'-moiety of the guanosine nucleophile in the Tetrahymena group I ribozyme. Biochemistry. 1999;38: 10958–75. 10460151 10.1021/bi990388e

[22] Karbstein K, Carroll KS, Herschlag D. Probing the Tetrahymena group I ribozyme reaction in both directions. Biochemistry. 2002;41: 11171–83. 12220182 10.1021/bi0202631

[23] Gleitsman KR, Herschlag DH. A kinetic and thermodynamic framework for the Azoarcus group I ribozyme reaction. RNA. 2014;20: 1732–46. 10.1261/rna.044362.114 25246656 PMC4201826

[24] Shan SO, Herschlag D. Dissection of a metal-ion-mediated conformational change in Tetrahymena ribozyme catalysis. RNA. 2002;8: 861–72. 12166641 10.1017/s1355838202020216 PMC1370303

[25] Shan S, Yoshida A, Sun S, Piccirilli JA, Herschlag D. Three metal ions at the active site of the Tetrahymena group I ribozyme. Proc Natl Acad Sci U S A. 1999;96: 12299–304. 10535916 10.1073/pnas.96.22.12299 PMC22911

[26] Wang SL, Karbstein K, Peracchi A, Beigelman L, Herschlag D. Identification of the hammerhead ribozyme metal ion binding site responsible for rescue of the deleterious effect of a cleavage site phosphorothioate. Biochemistry. 1999;38: 14363–78. 10572011 10.1021/bi9913202

[27] Christian EL. Identification and characterization of metal ion binding by thiophilic metal ion rescue Handbook of RNA biochemistry. 1 Weinheim: Wiley-VCH; 2005 p. 319–44.

[28] Frederiksen JK, Piccirilli JA. Identification of catalytic metal ion ligands in ribozymes. Methods. 2009;49: 148–66. 10.1016/j.ymeth.2009.07.005 19651216 PMC3470912

[29] Shan S, Kravchuk AV, Piccirilli JA, Herschlag D. Defining the catalytic metal ion interactions in the Tetrahymena ribozyme reaction. Biochemistry. 2001;40: 5161–71. 11318638 10.1021/bi002887h

[30] Lipchock SV, Strobel SA. A relaxed active site after exon ligation by the group I intron. Proc Natl Acad Sci U S A. 2008;105: 5699–704. 10.1073/pnas.0712016105 18408159 PMC2311373

[31] Cohn M, Shih N, Nick J. Reactivity and metal-dependent stereospecificity of the phosphorothioate analogs of atp in the arginine kinase reaction—Structure of the metal-nucleoside triphosphate substrate. J Biol Chem. 1982;257: 7646–9. 6282848

[32] Eckstein F. Phosphorothioate analogs of nucleotides—Tools for the investigation of biochemical processes. Angew Chem. 1983;22: 423–39.

[33] Christian EL. Identification and Characterization of Metal Ion Binding by Thiophilic Metal Ion Rescue In: Hartmann AB R. K, Schon A., Westhof E., editor. Handbook of RNA Biochemistry. Weinheim: Wiley-VCH; 2005.

[34] Kuo LY, Perera N, Tarpo S. Metal ion coordination to 2' functionality of guanosine mediates substrate-guanosine coupling in group I ribozymes: implications for conserved role of metal ions and for variability in RNA folding in ribozyme catalysis. Inorganica Chim Acta. 2004;357: 3934–42.

[35] Vicens Q, Cech TR. Atomic level architecture of group I introns revealed. Trends Biochem Sci. 2006;31: 41–51. 16356725 10.1016/j.tibs.2005.11.008

[36] Kuo LY, Piccirilli JA. Leaving group stabilization by metal ion coordination and hydrogen bond donation is an evolutionarily conserved feature of group I introns. Biochim Biophys Acta. 2001;1522: 158–66. 11779630 10.1016/s0167-4781(01)00327-x

[37] Strauss-Soukup JK, Strobel SA. A chemical phylogeny of group I introns based upon interference mapping of a bacterial ribozyme. J Mol Biol. 2000;302: 339–58. 10970738 10.1006/jmbi.2000.4056

[38] Mercer TR, Mattick JS. Structure and function of long noncoding RNAs in epigenetic regulation. Nat Struct Mol Biol. 2013;20: 300–7. 10.1038/nsmb.2480 23463315

[39] Chen W, Moore MJ. The spliceosome: disorder and dynamics defined. Curr Opin Struct Biol. 2014;24: 141–9. 10.1016/j.sbi.2014.01.009 24530854 PMC3987960

[40] Marcia M, Pyle AM. Visualizing group II intron catalysis through the stages of splicing. Cell. 2012;151: 497–507. 10.1016/j.cell.2012.09.033 23101623 PMC3628766

[41] Montange RK, Batey RT. Riboswitches: emerging themes in RNA structure and function. Annu Rev Biophys. 2008;37: 117–33. 10.1146/annurev.biophys.37.032807.130000 18573075

[42] Kuo LY, Cech TR. Conserved thermochemistry of guanosine nucleophile binding for structurally distinct group I ribozymes. Nucleic Acids Res. 1996;24: 3722–7. 8871550 10.1093/nar/24.19.3722 PMC146156

[43] Kim H. Characterization of the Guanosine Binding Process of the Twort Group I Ribozyme. Ph.D. Thesis, West Lafayette (IN): Purdue University; 2008.

[44] Chanfreau G, Jacquier A. An RNA conformational change between the two chemical steps of group II self-splicing. EMBO J. 1996;15: 3466–76. 8670849 PMC451911

[45] Hsieh J, Fierke CA. Conformational change in the Bacillus subtilis RNase P holoenzyme—pre-tRNA complex enhances substrate affinity and limits cleavage rate. RNA. 2009;15: 1565–77. 10.1261/rna.1639409 19549719 PMC2714742

[46] Martick M, Scott WG. Tertiary contacts distant from the active site prime a ribozyme for catalysis. Cell. 2006;126: 309–20. 16859740 10.1016/j.cell.2006.06.036 PMC4447102

[47] Zhuang X, Kim H, Pereira MJ, Babcock HP, Walter NG, Chu S. Correlating structural dynamics and function in single ribozyme molecules. Science. 2002;296: 1473–6. 12029135 10.1126/science.1069013

[48] Sripathi KN, Tay WW, Banas P, Otyepka M, Sponer J, Walter NG. Disparate HDV ribozyme crystal structures represent intermediates on a rugged free-energy landscape. RNA. 2014;20: 1112–28. 10.1261/rna.044982.114 24854621 PMC4114689

[49] Frank J, Gonzalez RL Jr. Structure and dynamics of a processive Brownian motor: the translating ribosome. Annu Rev Biochem. 2010;79: 381–412. 10.1146/annurev-biochem-060408-173330 20235828 PMC2917226

[50] Herschlag D, Eckstein F, Cech TR. Contributions of 2'-hydroxyl groups of the RNA substrate to binding and catalysis by the Tetrahymena ribozyme—an energetic picture of an active-site composed of RNA. Biochemistry. 1993;32: 8299–311. 7688572 10.1021/bi00083a034

[51] Kuo LY, Davidson LA, Pico S. Characterization of the Azoarcus ribozyme: tight binding to guanosine and substrate by an unusually small group I ribozyme. Biochim Biophys Acta. 1999;1489: 281–92. 10673029 10.1016/s0167-4781(99)00200-6

[52] Dai Q, Lea CR, Lu J, Piccirilli JA. Syntheses of (2')3'-15N-amino-(2')3'-deoxyguanosine and determination of their pKa values by 15N NMR spectroscopy. Org Lett. 2007;9: 3057–60. 17629287 10.1021/ol071129h

[53] Aurup H, Tuschl T, Benseler F, Ludwig J, Eckstein F. Oligonucleotide duplexes containing 2'-amino-2'-deoxycytidines: thermal stability and chemical reactivity. Nucleic Acids Res. 1994;22: 20–4. 8127651 10.1093/nar/22.1.20 PMC307740

[54] Shan SO, Narlikar GJ, Herschlag D. Protonated 2'-aminoguanosine as a probe of the electrostatic environment of the active site of the Tetrahymena group I ribozyme. Biochemistry. 1999;38: 10976–88. 10460152 10.1021/bi9903897

