# Supplementary figures and images for: Differential Assembly of Catalytic Interactions within the Conserved Active Sites of Two Ribozymes
Source: PLoS One. 2016 Aug 8;11(8):e0160457. doi: 10.1371/journal.pone.0160457 (PMC4976970; doi:10.1371/journal.pone.0160457)

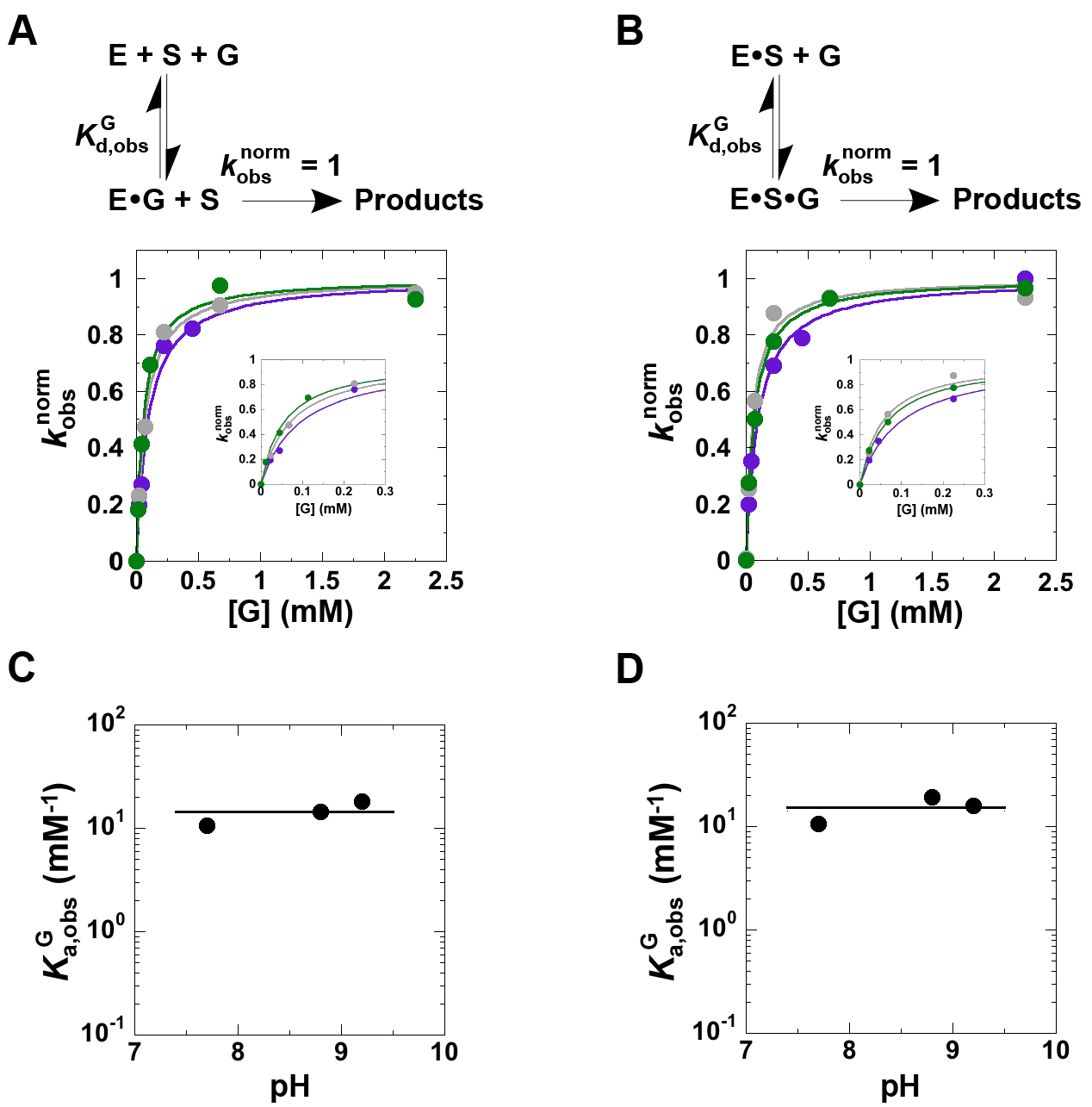

Supplement: S1 Fig — (A,B) G concentration dependence of the normalized rate of cleavage (kobsnorm) for E (A) and E•S (B). Measurements were made in a 15 mM Mg2+ background at pH 7.7 (purple), 8.8 (grey), and 9.2 (green).The lines are fits of the data from Eq 1 in Materials and Methods. (C,D) pH-dependence of G binding (Ka,obsG = 1/Kd,obsG) to E (C) and E•S (D) from the data shown in (A) and (B), respectively. The lines correspond to the mean of the measured G binding affinities to E (14.3 ± 4 mM-1) and E•S (15.2 ± 1 mM-1). Binding affinities are summarized in the S1 File. (TIF) [file pone.0160457.s001.tif]

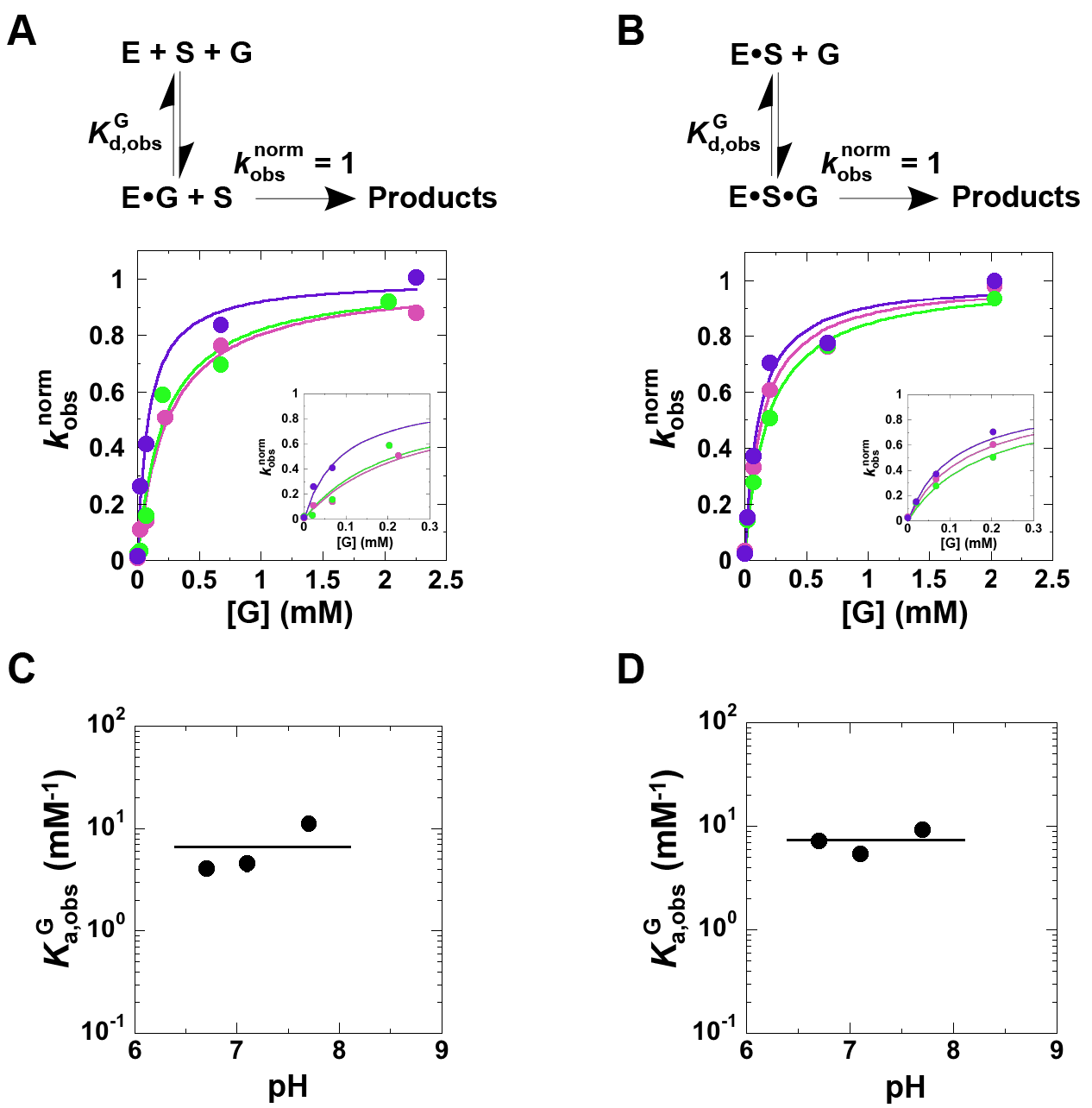

Supplement: S2 Fig — (A,B) G concentration dependence of the normalized rate of cleavage (kobsnorm) for E (A) and E•S (B). Measurements were made in the presence of 15 mM Mg2+ and 10 mM Mn2+ at pH 6.7 (pink), 7.1 (bright green), and 7.7 (purple). The lines are fits of the data from Eq 1 in Materials and Methods. (C,D) pH-dependence of G binding (Ka,obsG = 1/Kd,obsG) to E (C) and E•S (D) from the data shown in (A) and (B), respectively. The lines correspond to the mean of the measured G binding affinities to E (6.6 ± 4 mM-1) and E•S (7.3 ± 2 mM-1). Binding affinities are summarized in the S1 File. (TIF) [file pone.0160457.s002.tif]

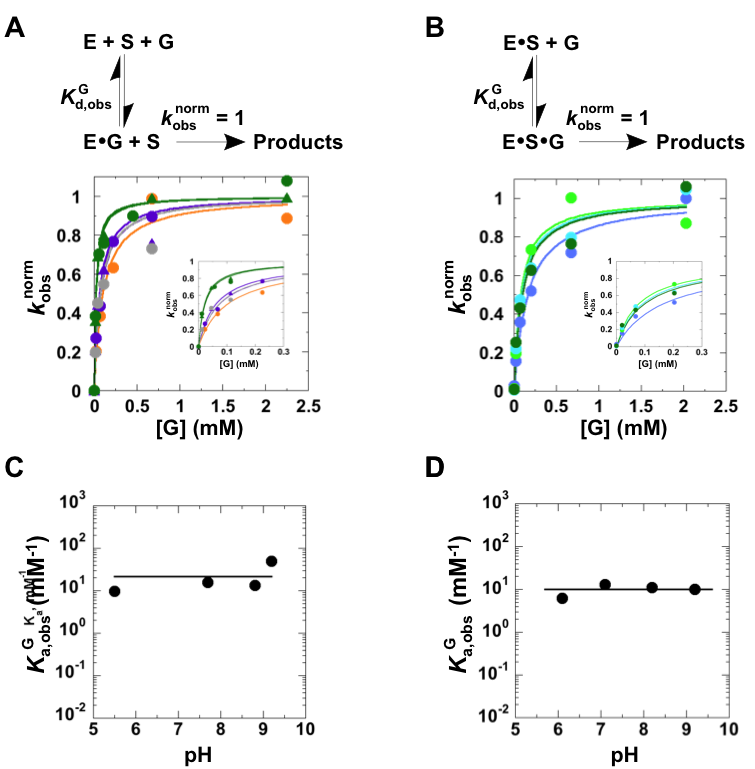

Supplement: S3 Fig — (A,B) G concentration dependence of the normalized rate of cleavage (kobsnorm) for E and E•S (B) in the presence of 100 mM Mg2+. Measurements were made at pH 5.5 (orange), 6.1 (blue), 7.1 (bright green), 7.7 (purple), 8.2 (bright blue), 8.8 (grey), and 9.2 (green). The different symbols (circles and triangles) at pH 7.7 and 9.2 denote two independent measurements made at these pH values. The line is a fit of the data from Eq 1 in Materials and Methods. (C,D) pH-dependence of G binding (Ka,obsG = 1/Kd,obsG) to E (C) and E•S (D) from the data shown in (A) and (B), respectively. The dashed lines are the mean of the measured affinities of G to E (21.9 ± 18 mM-1) and E•S (10.1 ± 3 mM-1). Binding affinities are summarized in the S1 File. (TIF) [file pone.0160457.s003.tif]

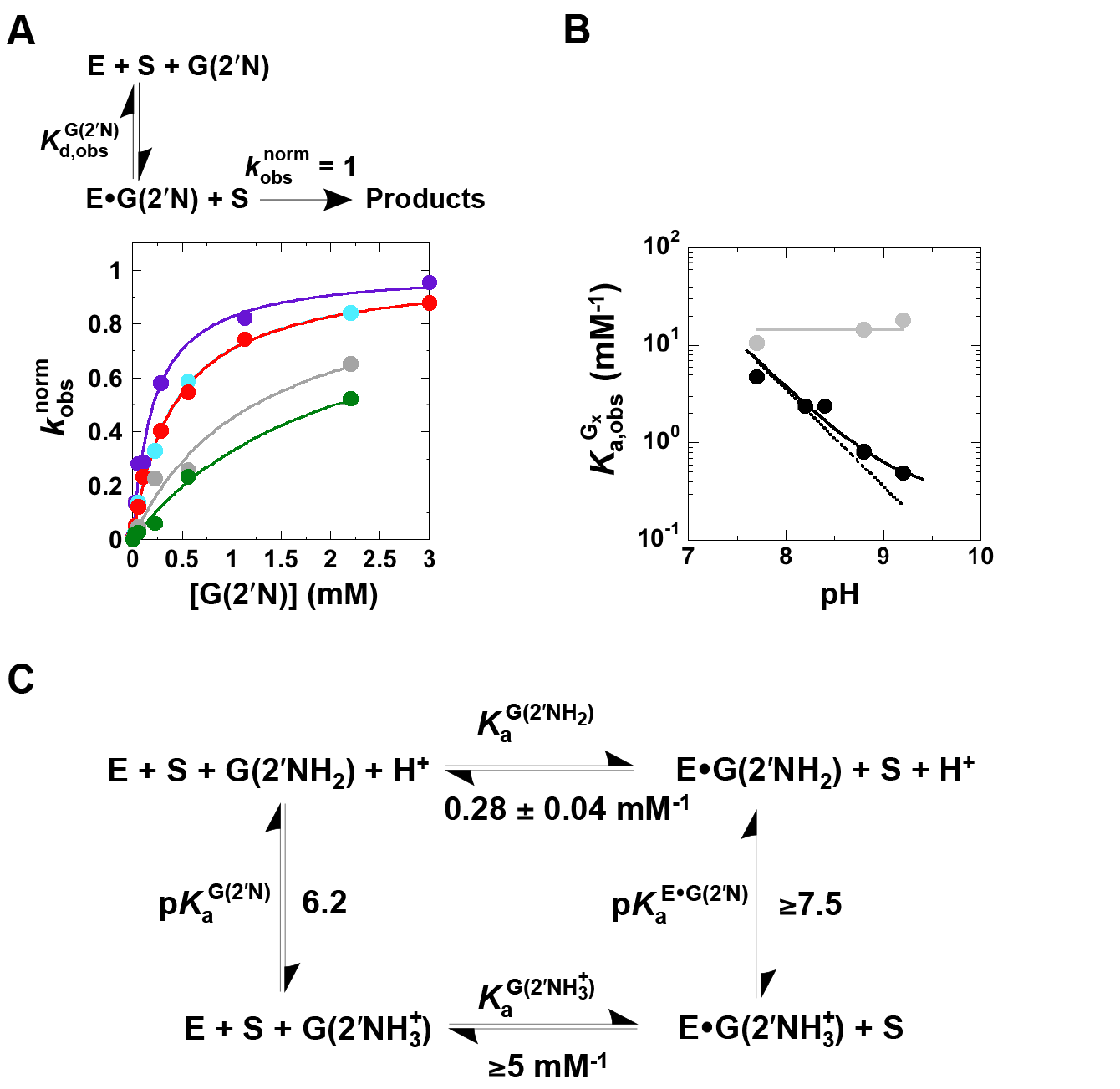

Supplement: S4 Fig — G(2′N) concentration dependence of the normalized rate of cleavage (kobsnorm) for E. Measurements were made at pH 7.7 (purple), 8.2 (bright blue), 8.4 (red), 8.8 (grey), and 9.2 (green). The lines are fits of the data from Eq 3 in Materials and Methods. (B) pH-dependence of G(2′N) (black) and G (grey) binding to E. The binding affinities (Ka,obsGx (= 1/Kd,obsGx) for G(2′N) and G were obtained from the data in (A) and S1A Fig, respectively. The solid line (black) is a fit of the G(2′N) data according to the model shown in (C) which describes binding of the -NH2 and -NH3+ forms of G(2′N) to E. For comparison, the data was also fit to a model in which the–NH2 form of G(2′N) does not bind to E (dotted line). The solid line (grey) is the average of the measured G affinities to E (14.3 mM-1; see S1C Fig). (C) Model for binding of G(2′NH2) and G(2′NH3+) to E. KaG(2'NH2) and KaG(2'NH3+) report binding of G(2′NH2) and G(2′NH3+) to E, respectively. A limit of >5 mM-1 was set for KaG(2'NH3+) since binding of G(2′N) did not level off at pH 7.7 ((Ka,obsG(2'N) = 5 mM-1). pKaG(2'N) and pKaE•G(2'N) are the equilibrium constants for deprotonation of G(2′NH3+) in solution and in E•G(2′N), respectively. The value of pKaG(2'N) was set to 6.2 [52–54], and pKaE•G(2'N) was determined by completing the thermodynamic cycle. Binding affinities are summarized in the S1 File. (TIF) [file pone.0160457.s004.tif]

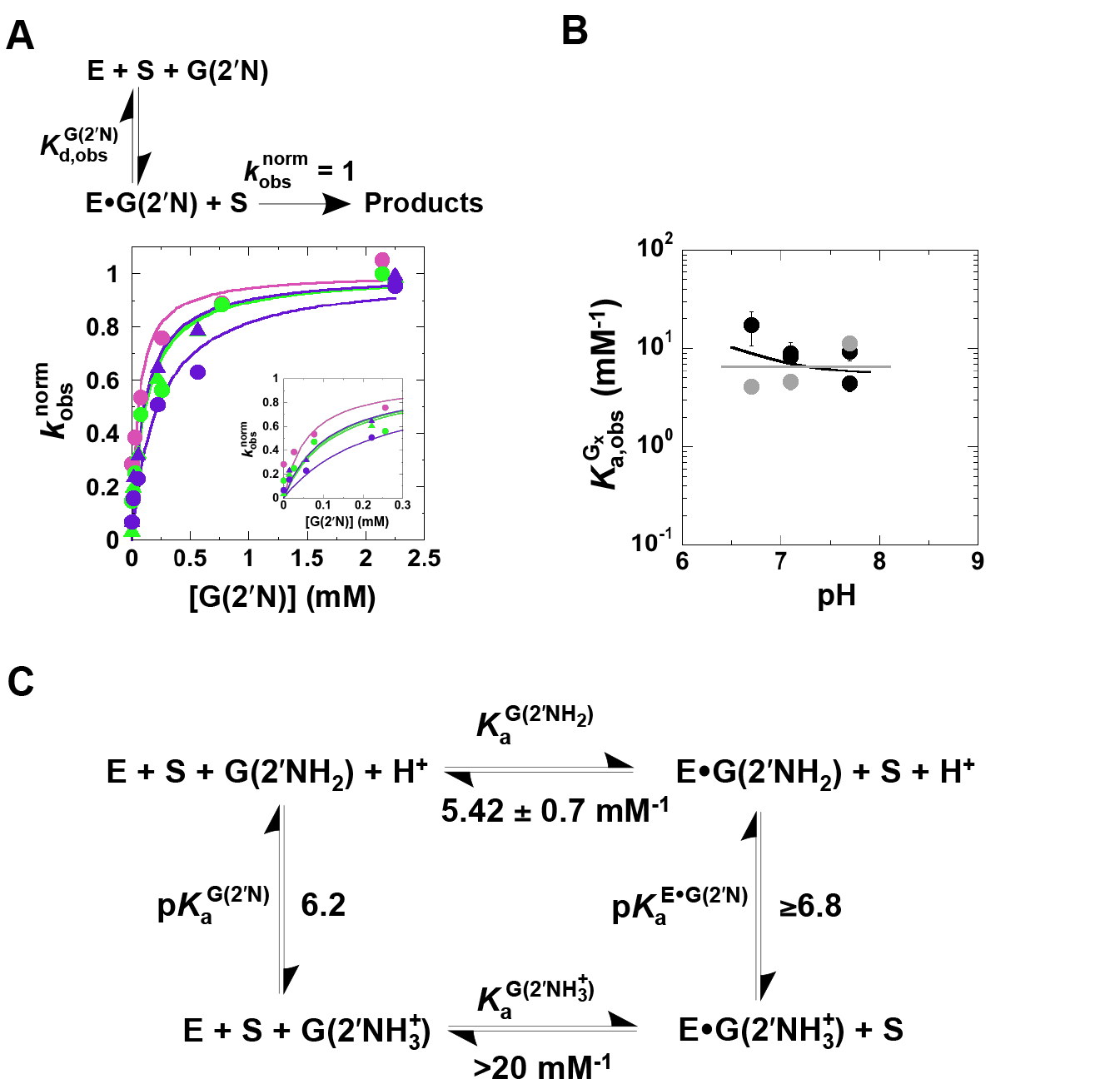

Supplement: S5 Fig — (A) G(2′N) concentration dependence of the normalized rate of cleavage (kobsnorm) for E in the presence of 15 mM Mg2+ and 10 mM Mn2+. Measurements were made at pH 6.7 (pink), 7.1 (bright green), and 7.7 (purple). The different symbols (circles and triangles) at pH 7.7 denote two independent measurements made at this pH. The lines are fits of the data from Eq 3 in Materials and Methods. (B) pH-dependence of G(2′N) (black) and G (grey) binding to E. The binding affinities (Ka,obsGx (= 1/Kd,obsGx) for G(2′N) and G were obtained from the data in (A) and S2A Fig, respectively. The black line is a fit of the G(2′N) data according to the model shown in (C) that describes binding of the -NH2 and -NH3+ forms of G(2′N) to E. The grey line is the average of the measured G affinities to E (6.6 mM-1; see S1C Fig). (C) Model for binding of G(2′NH2) and G(2′NH3+) to E. KaG(2'NH2) and KaG(2'NH3+) report binding of G(2′NH2) and G(2′NH3+) to E. A limit was set for KaG(2'NH3+) because binding of G(2′N) does not level off at the lowest pH measured. pKaG(2'N) and pKaE•G(2'N) are the equilibrium constants for deprotonation of G(2′NH3+) in solution and in E•G(2′N), respectively. pKaG(2'N) was set to 6.2 [52–54], and pKaE•G(2'N) was determined by completing the thermodynamic cycle. Binding affinities are summarized in the S1 File. (TIF) [file pone.0160457.s005.tif]

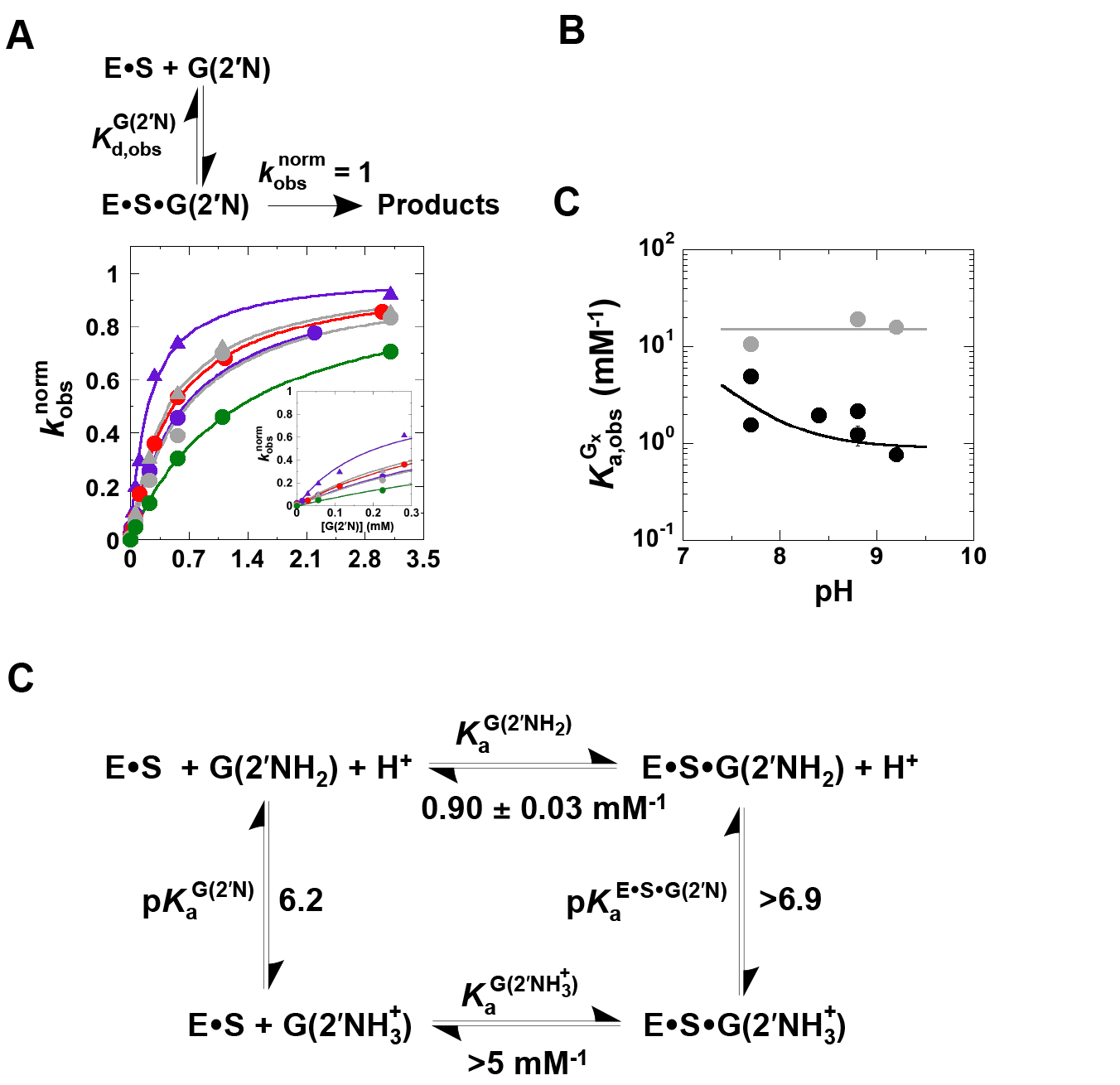

Supplement: S6 Fig — (A) G(2′N) concentration dependence of the normalized rate of cleavage (kobsnorm) for E•S in the presence of 15 mM Mg2+. Measurements were made at pH 7.7 (purple), 8.4 (red), pH 8.8 (grey), and pH 9.2 (green). The different symbols (circles and triangles) at pH 7.7 and 8.8 denote two independent measurements made at these pH values. The lines are fits of the data from Eq 3 in Materials and Methods. (C) pH-dependence of G(2′N) (black) and G (grey) binding to E•S. The binding affinities (Ka,obsGx (= 1/Kd,obsGx) for G(2′N) and G were obtained from the data in (A,B) and S1B Fig, respectively. The black line is a fit of the G(2′N) data according to the model shown in (C) which describes binding of the -NH2 and -NH3+ forms of G(2′N) to E•S. The grey line is the average of the measured G affinities to E•S (15.2 mM-1; see S1D Fig). (C) Model for binding of G(2′NH2) and G(2′NH3+) to E•S. KaG(2'NH2) and KaG(2'NH3+) report binding of G(2′NH2) and G(2′NH3+) to E•S, respectively. A limit of >5 mM-1 was set for KaG(2'NH3+) as binding of G(2′N) did not level off at pH 7.7 (Ka,obsG(2'N) = 5 mM-1). pKaG(2'N) and pKaE•S•G(2'N) are the equilibrium constants for deprotonation of G(2′NH3+) in solution and in E•S•G(2′N), respectively. pKaG(2'N) is 6.2 [52–54], and pKaE•S•G(2'N) was determined by completing the thermodynamic cycle. Binding affinities are summarized in the S1 File. (TIF) [file pone.0160457.s006.tif]

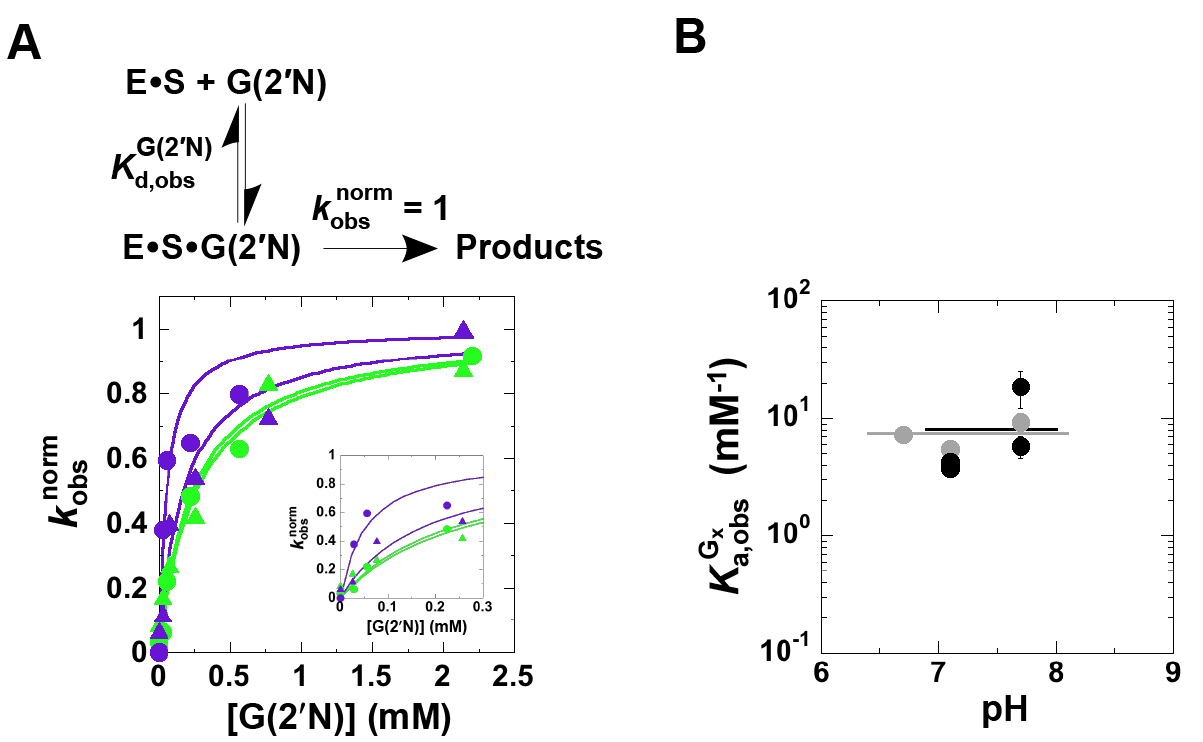

Supplement: S7 Fig — (A) G(2′N) concentration dependence of the normalized rate of cleavage (kobsnorm) for E•S in the presence of 15 mM Mg2+ and 10 mM Mn2+. Measurements were made at pH 7.1 (bright green) and pH 7.7 (purple). The different symbols (circles and triangles) at pH 7.1 and 7.7 denote two independent measurements made at these pH values. The lines are fits of the data from Eq 3 in Materials and Methods. (B) pH-dependence of G(2′N) (black) and G (grey) binding to E•S. The binding affinities (Ka,obsGx (= 1/Kd,obsGx) for G(2′N) and G were obtained from the data in (A) and S2B Fig, respectively. The lines are the average of the measured G (grey line, 7.3 mM-1; S2D Fig) and G(2′N) (black line, 8.1 ± 3 mM-1) affinities to E•S. The observation that varying the pH from 7.1 to 7.7 does not significant change Ka,obsG(2'N) suggests that the 2′-amino group exists in the -NH2 form above pH 7.1 [52–54]. (TIF) [file pone.0160457.s007.tif]

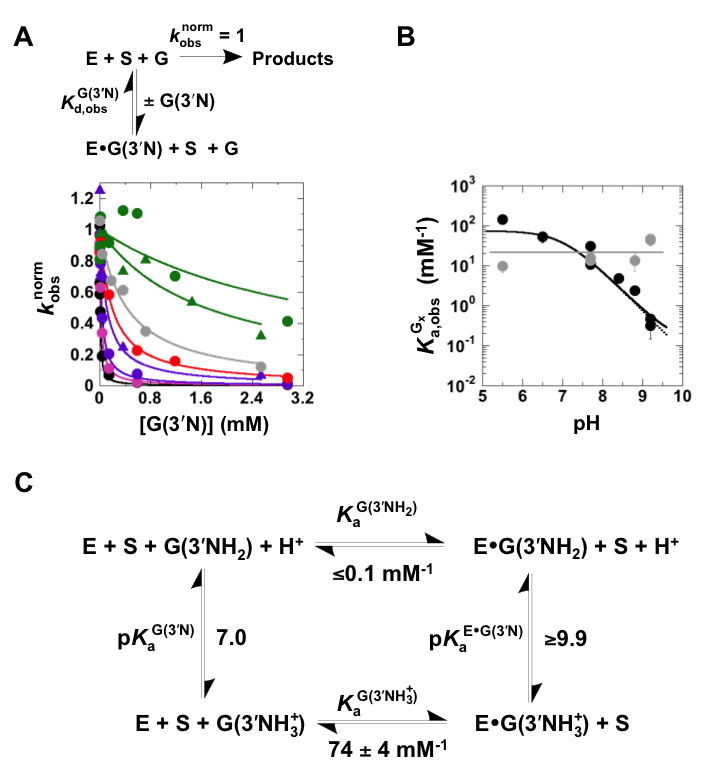

Supplement: S8 Fig — (A) G(3′N) inhibition of the normalized rate of cleavage (kobsnorm) of G with E and S in the presence of 100 mM Mg2+. Measurements were made at pH 5.5 (black), 6.5 (pink), 7.7 (purple), 8.4 (red), 8.8 (grey), and 9.2 (green). The different symbols (circles and triangles) at pH 9.2 denote two independent measurements made at this pH. The lines are fits of the data from Eq 2 in Materials and Methods. (B) pH-dependence of G(3′N) (black) and G (grey) binding to E. The binding affinities (Ka,obsGx (= 1/Kd,obsGx) for G(3′N) and G were obtained from the data in (A) and S3A Fig, respectively. The solid line (black) is a fit of the G(3′N) data according to the model shown in (C) which describes binding of the -NH2 and -NH3+ forms of G(3′N) to E. For comparison, the data was also fit to a model in which the–NH2 form of (3′N) does not bind to E (dotted line). The solid line (grey) is the average of the measured G affinities to E (21.9 mM-1; see S1C Fig). (C) Model for binding of G(3′NH2) and G(3′NH3+) to E. KaG(3'NH2) and KaG(3'NH3+) report binding of G(3′NH2) and G(3′NH3+) to E, respectively, and were obtained from the fit of the data in (B). The KaG(3'NH2) value is reported as a limit because binding of G(3′N) does not level off at high pH. pKaG(3'N) and pKaE•G(3'N) are the equilibrium constants for deprotonation of G(3′NH3+) in solution and in E•G(3′N), respectively. pKaG(3'N) is 7.0 [52], and pKaE•G(3'N) was determined by completing the thermodynamic cycle. Binding affinities are summarized in the S1 File. (TIF) [file pone.0160457.s008.tif]

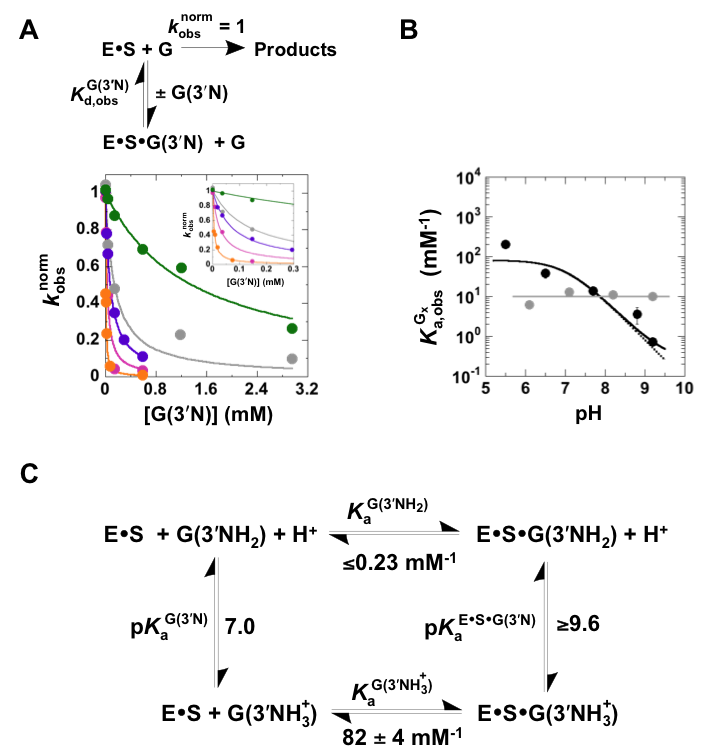

Supplement: S9 Fig — (A) G(3′N) inhibition of the normalized rate of cleavage (kobsnorm) of G with E•S in the presence of 100 mM Mg2+. Measurements were made at pH 5.5 (orange), 6.5 (pink), 7.7 (purple), 8.8 (grey), and 9.2 (green). The lines are fits of the data from Eq 2 in Materials and Methods. (B) pH-dependence of G(3′N) (black) and G (grey) binding to E•S. The binding affinities (Ka,obsGx (= 1/Kd,obsGx) for G(3′N) and G were obtained from the data in (A) and S3B Fig, respectively. The solid line (black) is a fit of the G(3′N) data according to the model shown in (C) which describes binding of the -NH2 and -NH3+ forms of G(3′N) to E•S. For comparison, the data was also fit to a model in which the–NH2 form of (3′N) does not bind to E•S (dotted line). The solid grey line is the average of the measured G affinities to E•S (10.1 mM-1; see S1D Fig). (C) Model for binding of G(3′NH2) and G(3′NH3+) to E•S. KaG(3'NH2) and KaG(3'NH3+) report binding of G(3′NH2) and G(3′NH3+) to E•S, respectively, and were obtained from the fit of the data in (B). The KaG(3'NH2) value is reported as a limit because binding of G(3′N) does not level off at high pH. pKaG(3'N) and pKaE•S•G(3'N) are the equilibrium constants for deprotonation of G(3′NH3+) in solution and in E•S•G(3′N), respectively. pKaG(3'N) is 7.0 [52], and pKaE•S•G(3'N) was determined by completing the thermodynamic cycle. Binding affinities are summarized in the S1 File. (TIF) [file pone.0160457.s009.tif]

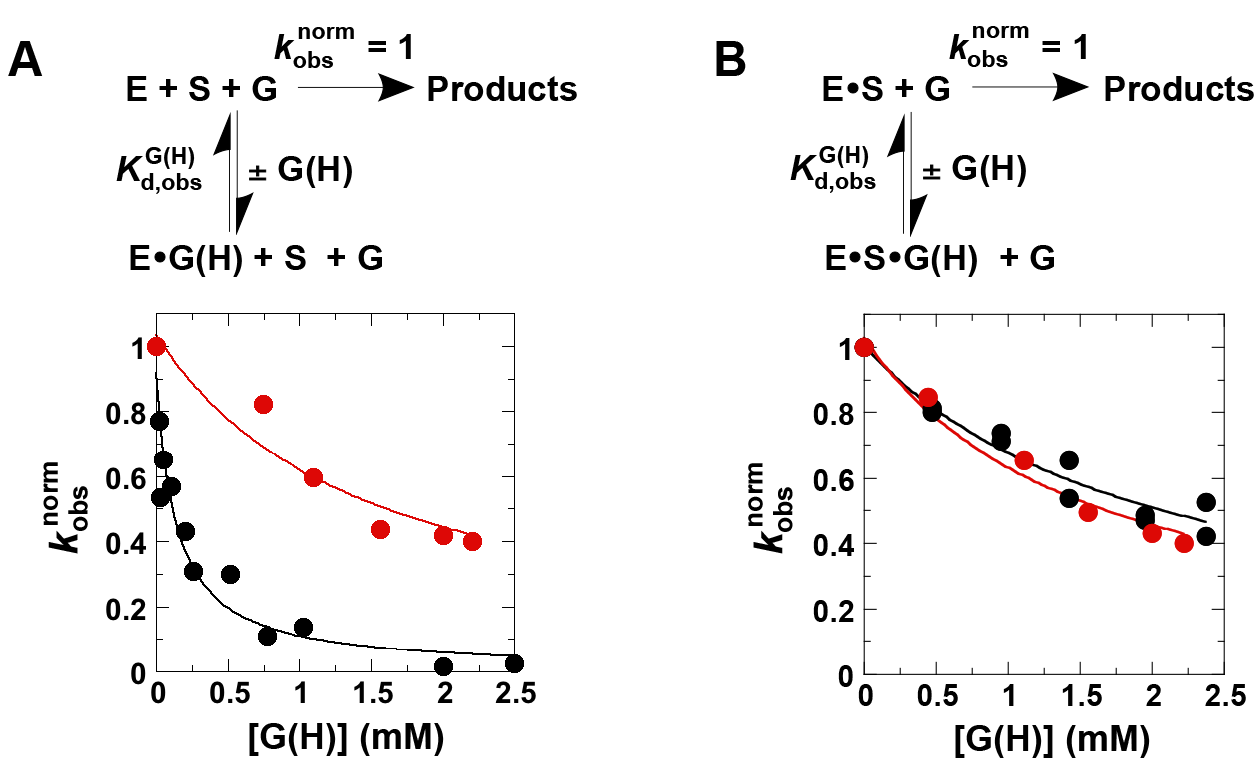

Supplement: S10 Fig — (A) G(2′H) (black) and G(3′H) (red) inhibition of the reaction of G with E and S (A) or with E•S (B). Measurements were made at pH 7.0 in the presence of 15 mM Mg2+. The data were analyzed as described in Materials and Methods. Binding affinities are summarized in the S1 File. (TIF) [file pone.0160457.s010.tif]
